# Functional analysis and transcriptional output of the Göttingen minipig genome

**DOI:** 10.1186/s12864-015-2119-7

**Published:** 2015-11-14

**Authors:** Tobias Heckel, Roland Schmucki, Marco Berrera, Stephan Ringshandl, Laura Badi, Guido Steiner, Morgane Ravon, Erich Küng, Bernd Kuhn, Nicole A. Kratochwil, Georg Schmitt, Anna Kiialainen, Corinne Nowaczyk, Hamina Daff, Azinwi Phina Khan, Isaac Lekolool, Roger Pelle, Edward Okoth, Richard Bishop, Claudia Daubenberger, Martin Ebeling, Ulrich Certa

**Affiliations:** Roche Pharmaceutical Research and Early Development (pRED), Roche Innovation Center Basel, Grenzacherstrasse 124, 4070 Basel, Switzerland; International Livestock Research Institute (ILRI), PO Box 30709, Nairobi, 00100 Kenya; Swiss Tropical and Public Health Institute (Swiss TPH), Socinstr. 57, CH 4002 Basel, Switzerland

**Keywords:** Comparative genomics, Transcriptional profiling, Pseudogene, Long non-coding RNA, Drug development and safety, Minipig

## Abstract

**Background:**

In the past decade the Göttingen minipig has gained increasing recognition as animal model in pharmaceutical and safety research because it recapitulates many aspects of human physiology and metabolism. Genome-based comparison of drug targets together with quantitative tissue expression analysis allows rational prediction of pharmacology and cross-reactivity of human drugs in animal models thereby improving drug attrition which is an important challenge in the process of drug development.

**Results:**

Here we present a new chromosome level based version of the Göttingen minipig genome together with a comparative transcriptional analysis of tissues with pharmaceutical relevance as basis for translational research. We relied on mapping and assembly of WGS (whole-genome-shotgun sequencing) derived reads to the reference genome of the Duroc pig and predict 19,228 human orthologous protein-coding genes. Genome-based prediction of the sequence of human drug targets enables the prediction of drug cross-reactivity based on conservation of binding sites. We further support the finding that the genome of *Sus scrofa* contains about ten-times less pseudogenized genes compared to other vertebrates. Among the functional human orthologs of these minipig pseudogenes we found HEPN1, a putative tumor suppressor gene. The genomes of *Sus scrofa*, the Tibetan boar, the African Bushpig, and the Warthog show sequence conservation of all inactivating HEPN1 mutations suggesting disruption before the evolutionary split of these pig species. We identify 133 *Sus scro*fa specific, conserved long non-coding RNAs (lncRNAs) in the minipig genome and show that these transcripts are highly conserved in the African pigs and the Tibetan boar suggesting functional significance. Using a new minipig specific microarray we show high conservation of gene expression signatures in 13 tissues with biomedical relevance between humans and adult minipigs. We underline this relationship for minipig and human liver where we could demonstrate similar expression levels for most phase I drug-metabolizing enzymes. Higher expression levels and metabolic activities were found for FMO1, AKR/CRs and for phase II drug metabolizing enzymes in minipig as compared to human. The variability of gene expression in equivalent human and minipig tissues is considerably higher in minipig organs, which is important for study design in case a human target belongs to this variable category in the minipig. The first analysis of gene expression in multiple tissues during development from young to adult shows that the majority of transcriptional programs are concluded four weeks after birth. This finding is in line with the advanced state of human postnatal organ development at comparative age categories and further supports the minipig as model for pediatric drug safety studies.

**Conclusions:**

Genome based assessment of sequence conservation combined with gene expression data in several tissues improves the translational value of the minipig for human drug development. The genome and gene expression data presented here are important resources for researchers using the minipig as model for biomedical research or commercial breeding. Potential impact of our data for comparative genomics, translational research, and experimental medicine are discussed.

**Electronic supplementary material:**

The online version of this article (doi:10.1186/s12864-015-2119-7) contains supplementary material, which is available to authorized users.

## Background

A critical step in drug development is the transition from the pre-clinical phase into clinical trials requiring experimental evidence that the drug candidate is reasonably safe in humans. Once a therapeutic indication, a biological effect, and a drug target or phenotype are defined, high-throughput screening and information-driven design techniques are employed to identify new chemical and biological starting points, which are further optimized to leads with the desired activity, e.g. as agonist or antagonist. Then, in line with regulatory requirements, appropriate animal models play a key role in pre-clinical development to ensure drug efficacy and safety. A wide range of animal models is considered for pharmacological efficacy studies including fish, rats, rabbits, and genetically engineered small- and large animal models such as mice and pigs [1–3]. For toxicological drug safety studies, however, the range of animal models is more restricted usually to a rodent and a non-rodent species since a well-defined battery of tests is required by guidelines of government agencies [4]. The most common animal species used to assess pre-clinical drug safety are rat as rodent species and beagle dogs and the Cynomolgus monkey (*Macaca fascicularis*) are accepted non-rodent models. In the last years the Göttingen minipig has gained growing attention as model for drug safety testing and translational medical research fueled by the RETHINK consortium [5]. These animals resemble many features of human anatomy, physiology, and biochemistry [5,6]. Furthermore, the status of pigs as livestock animals may relieve some ethical concerns associated with the use of dogs or primates as subjects for pre-clinical drug safety [7–9]. Multiple reciprocal crosses between Minnesota minipigs, Vietnamese potbelly pigs and German landrace pigs performed at the University of Göttingen gave rise in the 1990s to the founder minipigs of the Denmark based commercial breeding company Ellegaard [10]. Since then, minipigs with well documented breeding history are produced under highest hygienic and accredited animal welfare standards, thus fit for use in safety testing.

Following the advent of high-throughput genetic tools, genomic characterization of animal models has become indispensable for breeding purposes and has significantly improved interpretation of experimental data with respect to the translational value and relevance to human. In addition, genomic approaches significantly support rational species selection especially in the area of drug efficacy and safety. Primary sequence comparison of human drug target sequences with orthologs of animal models, for instance, allows prediction of drug cross-reactivity and responder species selection. Moreover, comparison of quantitative tissue expression profiles between humans and animal models allow the prediction or retrospective interpretation of tissue-specific pharmacodynamic and pharmacokinetic responses as well as modeling of drug exposure. In addition, identification and annotation of human orthologs enables design of species-specific analytical tools such as DNA microarrays or quantitative PCR assays.

Extensive breeding of domestic animals has generated significant phenotypic and genetic differences [11–14]. The first minipig genome sequence has recently been published by Vamathevan et al. [15] after the genomes of the domestic Duroc pig [11] and the Tibetan wild-boar [12]. According to Vamathevan et al., the Göttingen minipig genome lacks ~3000 protein coding genes when compared to the domestic pig and the Tibetian boar. A possible source for this significant discrepancy is the complex breeding history of the Göttingen minipig resulting in loss of non-essential genes. However, a more plausible explanation are different settings in algorithms for the prediction of human orthologous, multi-copy, and species-specific genes, or gaps and assembly inaccuracies, especially for a fully *de novo* assembled genome without chromosomal anchoring. Correct protein-coding gene predicitions in model organisms are crucial for translational medicine and therefore we generated a new chromosome anchored version of the minipig genome sequence termed Roche minipig genome. Using this assembly we identified about 2000 additional protein coding genes thereby approaching the gene count of *Sus scorfa* and the Tibetian boar. In addition we have used the Roche-genome combined with RNA-sequencing to design a minipig-specific microarray for transcriptional profiling in adult minipig tissues and during development from young to adult. Moreover, we describe minipig-specific lncRNAs and pseudogenes which are conserved in all available porcine genomes. The value of the minipig for translational research and as a model for drug safety assessment is discussed from a genomic perspective.

## Results

### The Roche minipig genome and comparative genomics

Recently, full-genome sequences of the Duroc farming pig [11], the Tibetan wild-boar [12], and the Göttingen minipig [15] were published. Using different methods, these genomes are predicted to harbor 21,640, 21,806, or 18,150 protein-coding genes for the Duroc pig, the Tibetan pig, and the Göttingen minipig, respectively. To explore this discrepancy we have generated a new minipig genome sequence using liver DNA isolated from a female minipig with documented breeding history from the commercial supplier Ellegaard. We used a combined Roche-454 and SOLiD sequencing approach and mapped all sequence reads on the latest version of the Duroc pig genome (*Sus scrofa* 10.2) which is the only available porcine genome assembly at the chromosome level. The mapping rate is ~93 % for Roche-454 reads and ~63 % for SOLiD reads resulting in total in ~20-fold genome coverage (Additional file 1: Tables S1 and Additional file 2: Table S2). For comparative genomics and gene identification we scanned our minipig genome together with the three other porcine genomes using a BLAST procedure [16]. 20,786 pig gene sequences from ENSEMBL were mapped to the Duroc pig genome with extremely high significance. From these 20,786 gene sequences 589 (2.8 %) could not be mapped on the Roche minipig genome draft (Additional file 3: Table S3); 441 of these 589 gene sequences are uncharacterized or not annotated genes. Therefore our Roche minipig genome scores a bit lower than the assemblies of the Tibetan pig (454 unmapped genes) and the *de novo* assembled minipig (449 unmapped genes), but on the other hand exhibits a slightly higher level of sequence identity of the mapped sequences (Additional file 4: Figure S1).

**Fig. 1:**
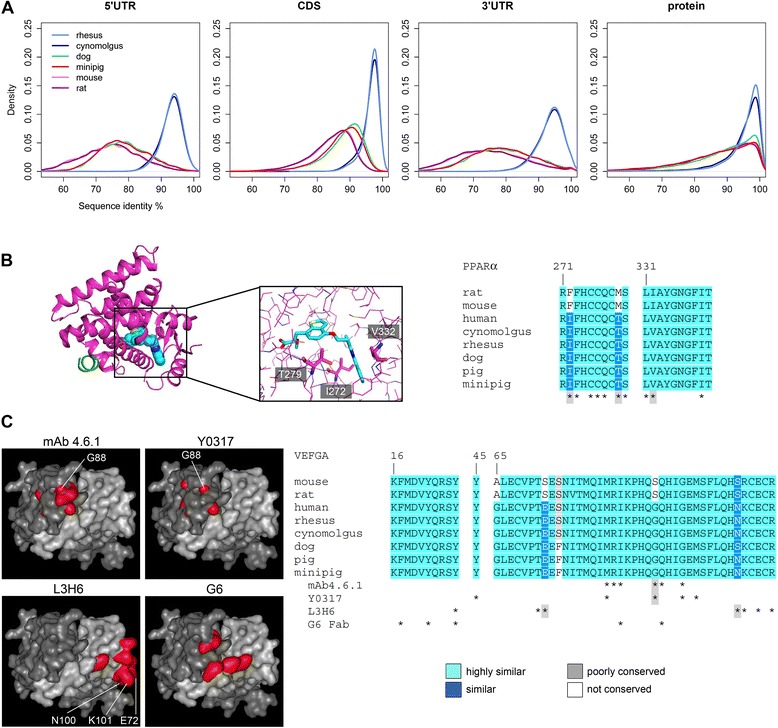
Multi-species sequence comparisons and assessment on drug binding. **a** Sequence identities between 1:1 orthologous transcripts and proteins of human, Rhesus macaque, Cynomolgus macaque, minipig, rat, and mouse. The 5’ UTR, CDS, and 3’ UTR of ~35,700 orthologous mRNAs (including splice variants) and of ~28,400 orthologous proteins were considered separately for the calculation of pairwise sequence identities in comparison to human. The relative number of 1:1 orthologous sequences was plotted against the sequence identities. Note that the peak sequence identities for the UTRs are significantly lower between humans and non-primates than for the coding regions. **b** Peroxisome proliferator-activated receptor alpha (PPARα) small molecule binding pocket analysis across multiple species. X-ray crystal structure of the ligand binding domain of human PPARα (magenta) with the dual PPARα/γ agonist aleglitazar (cyan) and with a 13-residue fragment of the SRC1 receptor co-activator motif 3 (green). Sequence alignments of PPARα orthologs from multiple species indicate that the contact residues (*) are fully conserved between human, macaques, and pigs while mouse and rat have sequence differences at three positions (P272, M279, I332) in comparison to the other species (I272, T279, V332). The inset shows the binding cavity in more detail and the non-conserved amino acids highlighted in a stick representation. PDB code: 3G8I. **c** Vascular endothelial growth factor (VEGF) epitope analysis for four different antibodies across multiple species. Depicted is the surface of human VEGF homodimer (light grey/dark grey) with residues relevant for antibody binding colored in red. Sequence alignments of VEGF orthologs from multiple species indicate for each antibody good conservation of contact residues (*) in human, macaques, and pigs, but not in rodents. Therefore cross-species reactivity is poor for mAb 4.6.1, the parent antibody of Avastin, and Y0317 (Lucentis) which are a product of immune response against hVEGF in mouse, and for the single-chain variable fragment (scFv) L3H6, targeting a different less conserved epitope. G6-Fab, derived from a synthetic antibody phage library, however shows good cross-reactivity due to full conservation of the functional epitope. PDB code: 1FLT

To explore the overall sequence conservation of minipig protein-coding genes compared to other major pre-clinical animal models and humans, sequence identity of minipig, dog, macaque and rodent transcriptomes with respect to human has been calculated for ~ 35,700 orthologous mRNAs (including splice variants) and ~ 28,400 proteins. As expected, the 5’- and 3’- untranslated RNA (5’ UTR, 3’ UTR) sequences (UTRs) exhibit lower identities than the coding sequences (CDS) and also lower identities for rodents with modes at ~74 %, than for macaques, with modes at ~94 %. For minipigs and dogs, UTR sequence identities were quite similar with modes at ~78 % (Fig. 1a). The CDS showed sequence identities of 88 % for rodents, 91 % for minipigs, 92 % for dogs and 98 % for macaques. At the protein level higher sequence identities with modes >97 % are calculated for all animal models.

For more reliable selection of an appropriate animal model for preclinical research primary sequence alignments should be complemented by the analysis of functional domains, small molecule binding pockets or epitopes targeted by therapeutic antibodies. For example, the X-ray co-crystal structure of the peroxisome proliferator-activated receptor alpha (PPARα) with the dual PPARα/γ agonist aleglitazar revealed its binding mode and 25 amino acids in contact with the ligand (distance ≤ 4.5 Å). These amino acids are fully conserved in human, macaques, dog, and minipig while in rodents three residues (I272F, T279M, V332I) are different (Fig. 1b). This difference likely explains the 45-fold lower receptor affinity of the agonist in mouse and rat compared to human [17] (Fig. 1b). Similarly, interspecies sequence conservation analysis of the residues forming the epitopes of therapeutic antibodies targeting soluble vascular endothelial growth factor (VEGF) provides possible explanation for experimentally measured differential affinity (Fig. 1c). Avastin (mAB 4.6.1) is used for cancer therapies, and Lucentis (Y0317) is the gold standard drug for treatment of age-related macular degeneration. L3H6 is an affinity-improved single-chain variable antibody fragment and G6 is a Fab fragment, both isolated from recombinant phage display libraries [18, 19]. Based on the 3D-models shown in Fig. 1c and affinity data, VEGF residue G88 is critical for high-affinity binding of Avastin and Lucentis. In rodents, the conserved glycine residue is changed to serine explaining the poor cross-reactivity of both therapeutic antibodies [18, 20]. The picture for L3H6 is more complex because high-affinity binding involves seven contact sites. Three out of these seven contact sites are changed in rodents (E72S, N100S, K101R) in agreement with the low cross-reactivity found experimentally. In G6, the contact sites on the VEGF dimer are conserved across all species included in the alignment, and sequence-based drug target assessment would predict cross-reactivity for all species as shown in Fig. 1c. As shown in the examples highlighted above, inter-species sequence conservation analysis can provide predictions of cross-reactivity of small molecule drugs or therapeutic antibodies given that the binding site residues are known.

### Disrupted genes/pseudogenes

Species differences in genes encoding human drug-targets are a critical parameter for species selection for translational research and preclinical drug development, especially when genes have become non-functional during evolution or breeding. Therefore the Roche minipig genome was scanned for non-functional, gene-like sequences. This analysis revealed 441 annotated pseudogenes in minipig consistent with published data for porcine genomes [11, 12, 15] (Additional file 3: Table S3). We further expanded our investigation for pseudogenes with protein-coding human orthologs that contain frame shift mutations or premature stop codons affecting protein translation and integrity in both the Duroc pig and our minipig genome, which are not represented in the pig protein NCBI-RefSeq database to reduce false positive discovery. This screen yielded 12 genes that are not functional in pigs as opposed to human (Additional file 5: Table S4). Among them, we found HEPN1, a tumor suppressor in hepatocellular carcinoma and pituitary somatotroph adenomas based on genetic evidence [21, 22]. In the minipig and Duroc farming pig the sequence of the HEPN1 gene shows a start codon mutation, two stop codons generating G/A mutations, and three frame-shifting insertions/deletions (Additional file 6: Figure S2). Since tumors in general and liver tumors in particular are of a comparatively low incidence in pigs [23–25], it is conceivable that HEPN1 function became dispensable early in porcine evolution. It is also possible that the inactive HEPN1 allele was introduced recently in commercial pig breeds as a result of extensive breeding over the last ~10,000 years [11]. Therefore we assembled the coding region of the HEPN1 gene from whole-genome-shotgun (WGS-) sequencing libraries from the African Warthog (*Phacochoerus africanus*) and two Bushpigs (*Potamochoerus larvatus*), because African and Eurasian pigs have evolved independently for at least ten million years without any reported contact or crosses [26, 27]. The DNA sequence and the position of all six HEPN1 mutations are identical in both African pig species and the Eurasian breeds (Additional file 6: Figure S2) indicating that HEPN1 was inactivated before the split into Eurasian and African pig species.

### Porcine specific transcripts

In primates, a global search in tissue expression databases yielded 131 primate-specific, polyadenylated, non-coding RNAs preferentially expressed in reproductive tissues [28]. Whilst microRNAs and small nucleolar RNAs are generally well-conserved in higher vertebrates long non-coding RNAs show high conservation within a genus [29]. To identify porcine specific transcripts in domestic pigs we followed the experimental strategy by Tay et al. [28] and sequenced minipig testis RNA on three different sequencing platforms (Additional file 7: Figure S3). Minipig sequences were assembled and mapped on several vertebrate genomes to end up with 133 non-coding porcine-specific transcript candidates. Because primate specific transcripts are highly conserved across many primate species, we mapped our 133 lncRNA sequences to all the available porcine genome sequences. In the Roche minipig genome 130 out of 133 loci are detected in the genome with 100 % sequence identity followed by the Duroc pig (127 out of 133) and the Tibetan boar (124 out of 133; Additional file 8: Figure S4). In the Göttingen minipig genome, 126 lncRNAs are detected matching 100 % and about 15RNAs are detected with partial homology. For example, the lncRNA G8MPFOXO1AQL9 appears to be absent in the genome of Duroc, the Tibetan boar and our minipig whilst present in the Göttingen minipig genome from Vamathevan et al., and yet it is present in three independent testis RNA sequencing libraries. For the African Warthog and the Bushpig, we compared all available reads coming from our WGS experiment to the sequences of all 133 minipig lncRNAs to cope with lack of an assembled genome. One hundred twenty one lncRNAs had perfect matches with reads from these random libraries above an arbitrary detection threshold (Additional file 9: Table S5). This reversed mapping approach does not allow a conclusive statement about sequence conservation except that all mapping reads had at least 95 % identity (data not shown). We conclude that the pig family possesses like primates specific transcripts with comparable abundance and considerable sequence conservation in Eurasian and African species.

### Comparative analysis of gene expression in human and minipig tissues

**Fig. 2:**
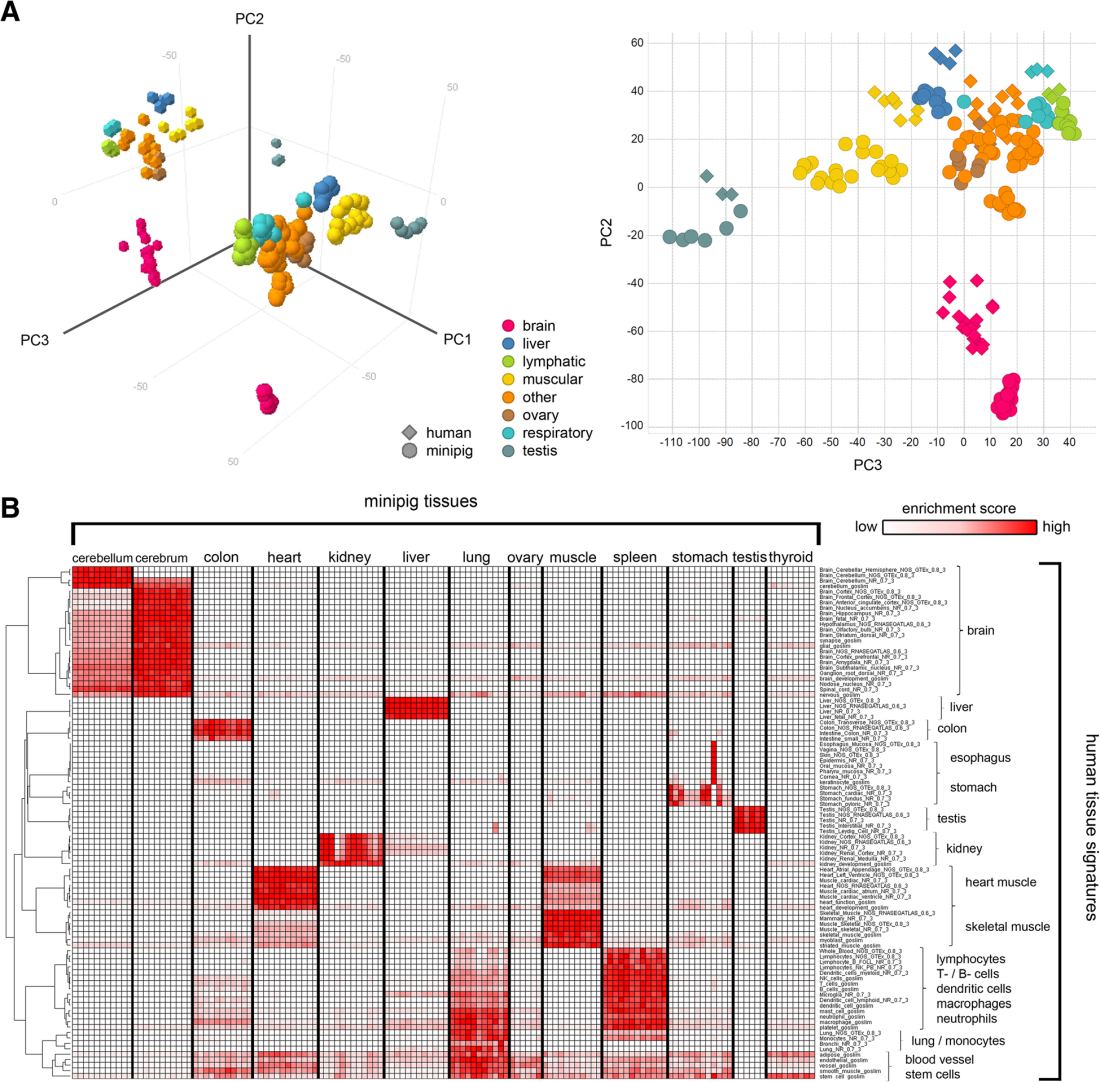
Global Comparison of human and minipig tissue expression profiles. **a** Principal component analysis of whole transcriptome expression profiles of human (*n* = 3 - 9) and minipig tissues (*n* = 9 - 12) based on 16’032 orthologous genes. A common set of 13 tissue types is represented for each species. Tissues types were colored according to organ systems. Principal component 1 accounts for 29 %, principal component 2 for 11 %, and principal component 3 for 6 % of the variation of the data set. Related tissues cluster together between species. **b** Comparison of minipig whole transcriptome expression profiles to human tissue signatures. Enrichment scores are indicated by a relative color scale (by row); with red representing high similarity and white no similarity of minipig gene expression profiles to human tissue specific gene expression signatures

Gene expression profiles across multiple tissues allow the prediction of shared functional properties of animal and human tissues including prediction of tissue-specific drug responses. For a comprehensive analysis of the minipig transcriptome in one year-adults, we constructed minipig specific gene expression microarrays to build a minipig gene expression database with a set of 18 tissues with high relevance for biomedical research (aorta, cerebrum, cerebellum, colon, duodenum, gall bladder, heart, jejunum, kidney, liver, lung, ovary, skeletal muscle, spleen, stomach, testis, thyroid glands, and urinary bladder). We used 12 biological replicates per tissue (six male and six female) to support statistical data analysis with concomitant detection of inter-individual variability. Inspection of the first three principal components, accounting for 29 % of the observed variability in the expression profiles of 18 tissues, revealed that biological replicates and biologically related tissues from the same organ system cluster together (data not shown). Interestingly, brain and testis tissue transcriptomes were most different to the other minipig tissues in the principal component analysis (PCA). To compare minipig tissue expression profiles to their human counterparts, we used a high quality human microarray dataset with 13 overlapping tissues and 16,032 shared genes [30]. Both datasets were normalized first within and second across both species with the global rank invariant set normalization (GRSN) method to reduce systematic distortions in microarray data [31]. The PCA of the GRSN normalized expression data showed that principal component 1 separates the two species whilst principal component 2 and principal component 3 show very similar clustering of human and minipig tissue transcriptomes reflecting also the special complexity of brain and testis in both species (Fig. 2a). We extracted signature genes with significant enrichment in specific tissues from several human tissue expression data sets available in public databases. These signatures allowed the determination of minipig tissue transcriptomes with gene expression profiles similar to human. Using this approach combined with unsupervised clustering demonstrates high similarity of human and minipig tissues at the transcriptional level (Fig. 2b). Moreover, the conserved tissue specific gene signatures in minipig support similarity of core organ functions to human together with similarities in metabolism and physiology.

### Hepatic drug metabolism in minipig and human

**Fig. 3:**
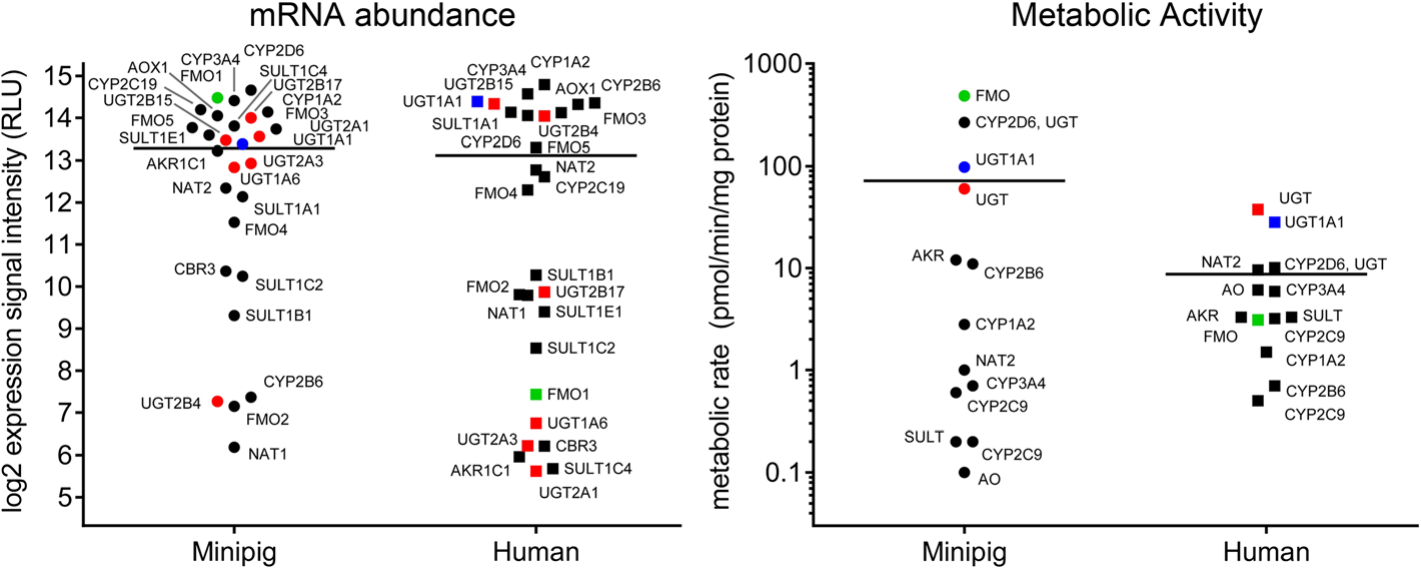
Correlation of transcript abundance and metabolic activity for major drug metabolizing enzymes in human and minipig liver. Liver mRNA abundance of all major phase I and II drug metabolizing enzymes was determined with minipig and human microarray data (left panel). Metabolite formation rates (pmol/min/mg protein) were determined for phase I and II enzyme substrates for human and minipig primary hepatocyte suspension cultures (right panel). Transcript abundance and metabolic activities of human and minipig major cytochrome p450 enzymes (CYPs), aldoketoreductases (AKR/CR), aldehyde oxidase (AOX), N-acetyltransferases (NATs), and sulfotransferases (SULT) are shown as black dots. UDP-glucuronosyltransferase (UGT) family members are shown as red dots, UGT1A1 as separate blue dots, and flavin-monooxygenase 1 (FMO1) as green dots. Liver mRNA expression levels show higher expression for FMO1, UGTs, SULT1C4, and SULT1E1 in minipig as compared to human. The UGT family expression data is represented by four subtypes detected by isoform specific probes. All major phase I CYPs show very similar expression levels in both species. Higher UGT, AKR, and FMO1 enzyme activities compare well with higher liver gene expression levels in minipig. Bars present average transcript abundance and metabolic activity

Several studies indicate that the activity of drug metabolizing enzymes and liver physiology is similar between pigs and humans [32–34]. In general, the mRNA expression levels of major cytochrome p450 enzymes (CYPs), 12 aldoketo-/carbonyl-reductases, flavin-containing monooxygenases (FMOs), aldehyde oxidase 1 (AOX1), seven major UDP-glucuronosyltransferases (UGTs), two N-acetyltransferases (NAT1, NAT2), and five sulfotransferase 1 family members (SULTs) are similar in humans and minipigs with the exception of the inducible CYP2B6 isoform (Fig. 3, left panel). In contrast, mRNA expression levels of the UGT-isozymes UGT1A6, UGT2A1, UGT2A3, and UGT2B17 and the rest of the selected drug metabolizing enzymes (FMO1, aldoketoreductase AKR1C1, carbonylreductase CBR3, sulfotransferases SULT1C4 and SULT1E1) are significantly higher in minipig liver compared to human. In order to correlate mRNA abundance with enzymatic activity, we measure the metabolic activity of a subset of enzymes for which specific reporter substrates are available (midazolam [CYP3A4], dextromethorphan [CYP2D6, UGT], diclofenac [CYP2C9], tolbutamide [CYP2C9], buproprion [CYP2B6], tacrine [CYP1A2], benzydamine [FMO1/3], daunorubicin [AKR/CR], O6-benzlyguanine [AO], sulfamethazine [NAT2 ], SN-38 [UGT1A1] and 7-hydroxycoumarin [UGT,SULT]). For the selected set of enzymes, the mRNA expression levels and the metabolic activity correlated well between human and minipig liver with some exceptions (Fig. 3). The activities of CYP3A4 and CYP2C9 for instance are lower in minipig liver whilst the activities of CYP2B6 and CYP1A2 are elevated. Proportional to differential mRNA expression, the metabolic formation rate of benzydamine N-oxide catalyzed by FMO1 and FMO3 [35] is 153-fold higher in minipig liver. Based on the examples above, mRNA expression levels might serve as surrogate to predict the metabolism of human drugs in minipig liver provided that the catalyzing enzyme is known.

### Tissue specific changes in gene expression from young to adult

**Fig. 4:**
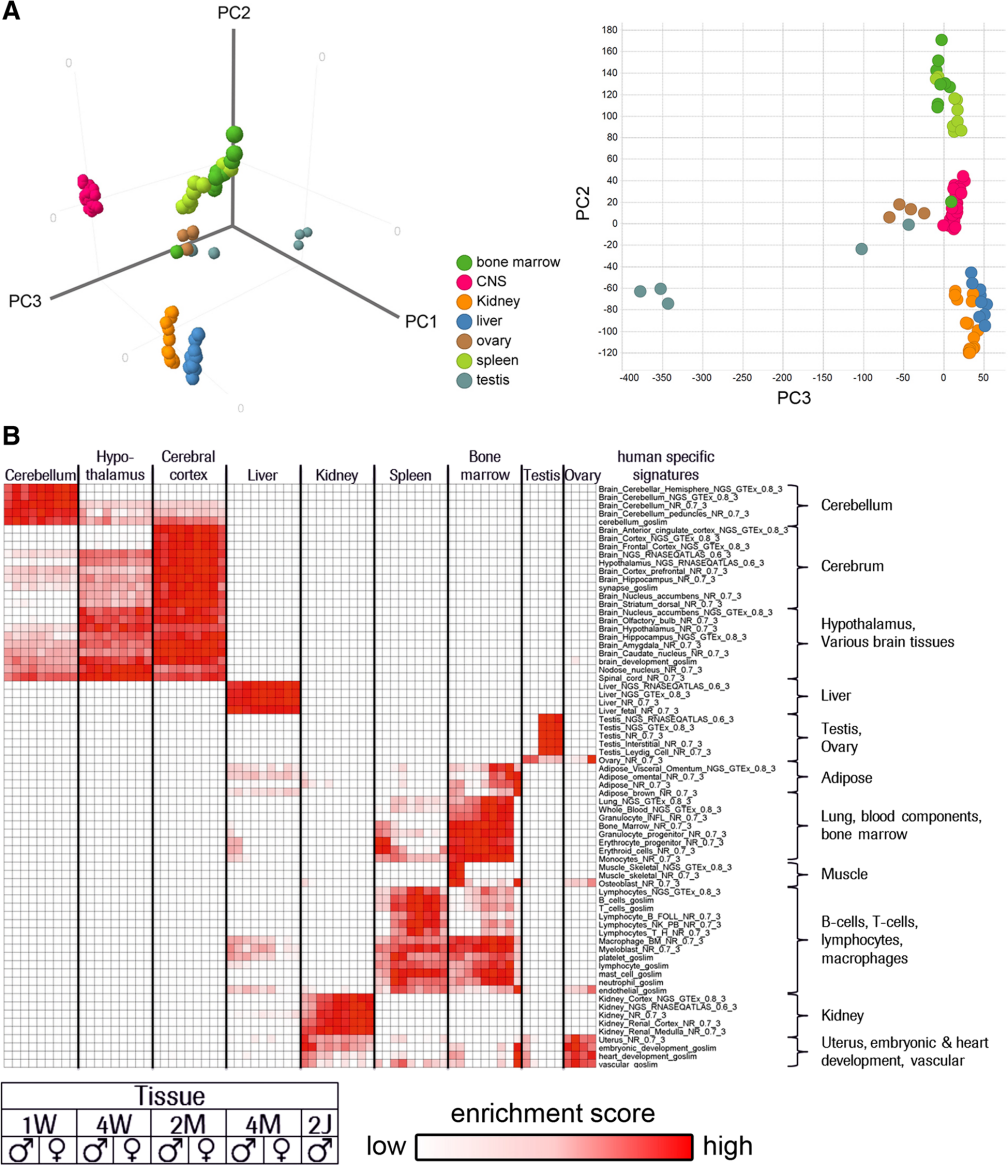
Gene expression developmentsin minipig organs relevant for juvenile toxicity from young to adult. **a** Principal component analysis of whole transcriptome expression profiles of minipig tissues based on 17,254 genes. Principal component 1 accounts for 21.8 %, principal component 2 for 12.4 %, and principal component 3 for 11.3 % of total variation of the data set. **b** The heat map shows enrichment scores for human tissue expression signatures (red = high; white = low) in minipig tissues from young to adult. With exception of reproductive organs pairwise male and female data points for 1 week, 4 weeks, 2 months, 4 months and adult are shown

Studies in juvenile animals are needed when existing data from animals and humans are insufficient to predict efficacy and safety of drugs in children. Minipigs have favorable biological characteristics for juvenile toxicity studies such as a relatively large litter size of 4-6 piglets/sow, cross-fostering of randomly allocated piglets for genetically heterogeneous group compositions, a “brain growth spurt” similar to neonatal humans, rapid growth and development, sexual maturity at an early age, and easy handling of piglets. The developmental stage of a 6 year old human child, for example, corresponds to a minipig at the age of about 2 months [36]. Since many organ systems such as brain or the reproductive system are still developing in pediatric populations, they may be prone to pharmacodynamic effects or toxicities not evident in studies in adults [37]. Apart from the features above that are in favor of the minipig as model for juvenile studies, nothing is known at the molecular level at which stage after birth organ development is complete. Especially data regarding the expression of drug targets, drug metabolism pathways or maturation of the immune system during development are critical for rational study design. Therefore, we performed microarray based tissue expression profiling in 9 tissues relevant for juvenile toxicity assessment (cerebellum, hypothalamus, cerebral cortex, liver, kidney, spleen, bone marrow, testis, and ovary) from shortly after birth to adulthood (1 week, 4 weeks, 2 months, 4 months and 2 years). Based on literature, these developmental stages generally correspond in humans to a newborn, a 2 year old toddler, a child at the age of 6 years, an adolescent at the age of 14 years/around puberty and a sexually mature adult [38]. To compare tissue expression profiles to each other during postnatal development, we used PCA. The entire dataset shows clustering of tissue samples regardless of the developmental stage suggesting early completion of the transcriptional programs in the minipig (Fig. 4a). According to this analysis only testis shows significant maturation during development which coincides with the onset of male fertility two months after birth (pers. communication with Ellegaard and Ellegaard newsletter 43, spring 2015). This analysis further confirms that the specialization of the testis transcriptome in adults as indicated in our PCA analysis (Additional file 6: Figure S2A and Additional file 8: Figure S4A) is indeed related to development. Analogous to the multi-tissue analysis in adults (Fig. 2b) we have used human tissue specific signatures for further examination of minipig tissue transcriptome maturation (Fig. 4b). This high-level analysis shows first of all no gender segregation with exception of reproductive tissues, and secondly and perhaps more important, that the majority of gene expression programs in the tissues analyzed are completed shortly after birth except for testis, bone-marrow, and spleen. For example liver transcriptomes share during all time points analyzed tissue specific expression features with minipig and human adults. Moreover, the expression of hepatic genes for drug metabolism and excretion - phase I and phase II enzymes and drug transporters - reaches adult levels one week after birth (data not shown). This finding is consistent with limited public data on the developmental expression of some minipig and human CYP450 enzymes and drug transporters suggesting that the major switch-on appears to occur in minipig like in humans shortly after birth [39, 40]. Compared to most pathways, maturation of the immune system is delayed and occurs between four and eight weeks in spleen and bone-marrow based on human B- and T-cell signatures (Fig. 3b). In addition, this analysis confirms that testis specific genes are expressed two months after birth. In summary, this analysis of gene expression profiles provides the result that the transcriptional program in minipigs is basically completed at the age of four weeks and underlines the advanced state of development in many organ systems at birth. Based on this outcome, we conclude that molecular information from developing piglets further strengthens the interpretation and translatability of juvenile toxicity studies in minipigs.

### Identification of genes with variable expression in minipig and human tissues

**Fig. 5:**
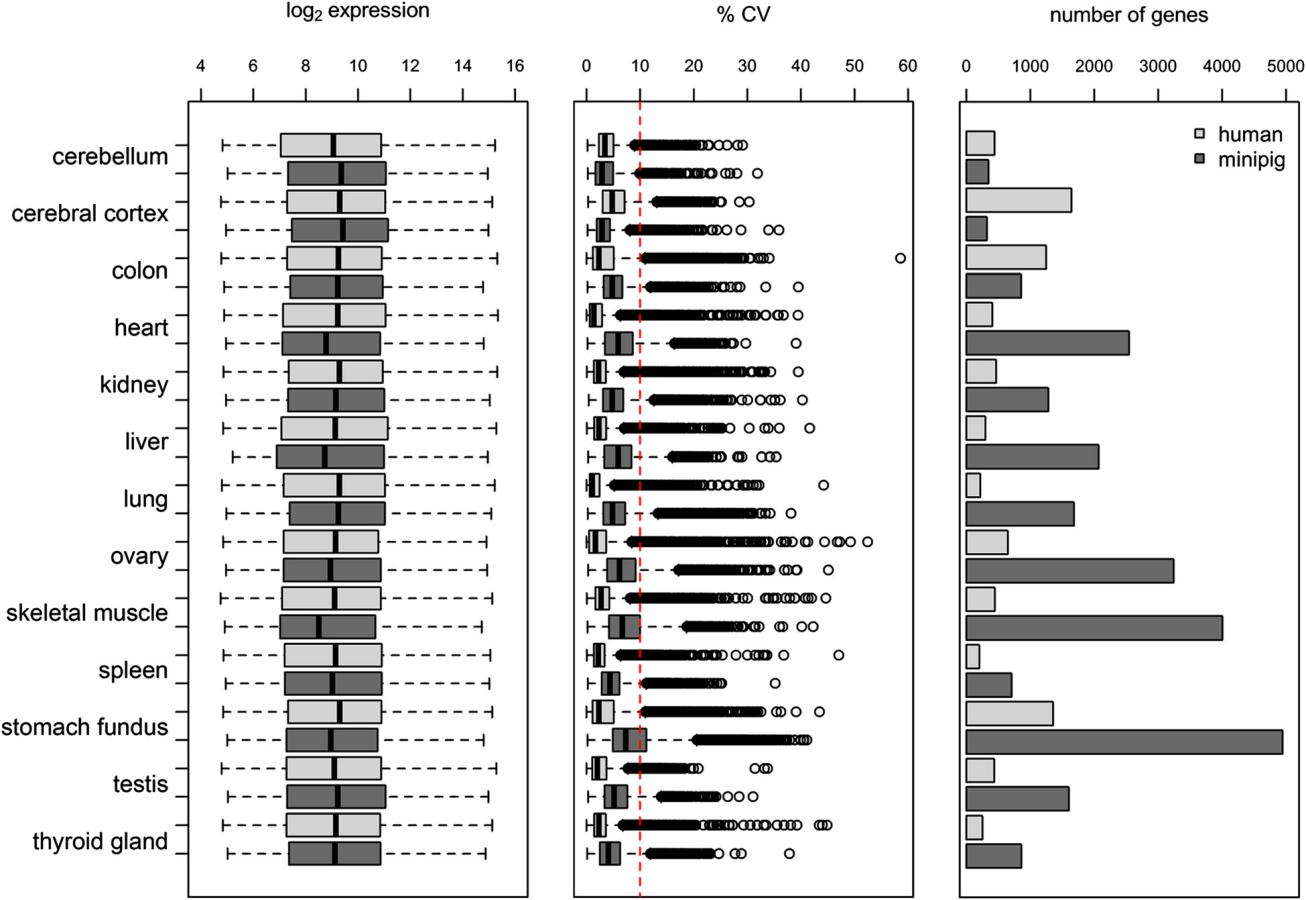
Global variability of tissue gene expression profiles in human and minipig. The left bar chart displays the average log_2_ expression levels, the middle bar chart the corresponding coefficient of variation (CV) profiles, and the right bar chart high variance genes per tissue and species. High variance genes were identified from transcriptome-wide expression signals using a 10 % CV cutoff. The number of high variance genes per tissue and species indicates that cerebellum is the least variable tissue in expression in both species and stomach is the most variable. Notably, gene expression in minipigs appears in general more variable per tissue than in humans

We have previously shown in *Macaca fascicularis* liver tissue that about 4 % of the genes show highly variable expression independent of transcript abundance [41]. To guide translational research we compared the number of genes with variable expression in 13 minipig tissues from 9 to 12 animals with matching human microarray data across 16,032 shared genes (Fig. 4) [30]. Both datasets were GRSN normalized resulting in a consistent distribution of the mean expression values ensuring comparability within and across species for each tissue. To identify expression variability the coefficient of variation (CV) was computed for each gene per tissue and species. Genes were considered as being highly variable with a CV > 10 % (Fig. 5). Using this measure of variance, we find that the number of highly variable genes across 13 tissues is low in both species in cerebellum with about 2–3 % of all genes and high in stomach with up to 30 % of all genes in minipig. Moreover, ten out of 13 tissues harbor at least 3-fold more highly variable genes in minipigs than in humans, which is surprising in light of low genetic exchange, health monitoring and contolled environment at the vendor’s breeding center. This difference is statistically highly significant (*p* < 0.001) as determined by a proportionality test and independent of gender except for reproductive organs. An enrichment analysis of these lowly-constrained genes for Gene Ontology (GO) terms reveals certain functional roles for kidney, ovary, and stomach. For kidney, the sodium/glucose transporter of the transmembrane transport process (GO:0055085, *p* < 10^−8^), the secreted kidney protease renin, and the peptide hormone angiotensin are highly variable in both species. This finding points towards a link between inter-individual differences in diet and the Renin-Angiotensin-Aldosterone-system controlling the expression of sodium channels [42]. For ovary, we found that the annotations for the regulation of growth (GO:0040008, *p* < 10^−6^ ) and for the steroid biosynthetic process (GO:0006694, *p* < 10^−5^) are most prominently enriched, indicating inter-individual differences along the growth hormone axis and the menstrual cycle [43, 44]. Finally for stomach, the cell adhesion process (GO:0007155, *p* < 10^−7^) seems to be most variable between individuals. In summary, gene expression variability is considerably different between organs and should be taken into account for study design. For example, if a new drug target belongs to the category of highly variable genes, larger animal groups are granted to discriminate drug related findings from inter-individual variability. Genes with stable expression on the other hand may allow for smaller experimental groups compliant with the 3R principles of animal welfare [45].

## Discussion and conclusions

In this work we present a comprehensive analysis of the transcriptional output of the minipig genome covering developmental and tissue specific gene expression combined with genome based detection of private pseudogenes and non-coding transcripts absent in the currently available genomes of eukaryotic organisms.

As a basis for this study, we have re-sequenced the minipig genome because any type of gene expression data rely on correct annotation and specificity of tools and assays. Apart from phenotype, most characteristics such as metabolism or physiology are shared between minipig and the Duroc pig and therefore we expect no major genomic differences caused by breeding or environmental adaptation as seen for the olfactory or hypoxia genes in the Tibetan wild boar [12]. For the Duroc and Tibetan pigs, 21,640 or 21,806 protein-coding genes are predicted, respectively, which is in good agreement [11, 12]. In contrast, Vamathevan et al. predict for their *de novo* assembled minipig genome 18’150 protein-coding genes [15]. To shed light on this discrepancy with potential impact for research we sequenced the genome of a female Göttingen minipig by combining Roche-454 long read pyrosequencing technology for contig and scaffold assembly to the Duroc reference genome and SOLiD short read technology to increase sequence coverage. In the final Roche minipig genome we identified ~2000 additional protein coding genes resulting in a final gene count of 20,197 thereby approaching the gene count in Duroc and Tibetan pigs (Additional file 3: Table S3). Furthermore, our analysis revealed 441 minipig pseudogenes which is a low number but consistent across different pig species [11,12,15]. In contrast, the genomes of *Mus musculus* and *Homo sapiens* contain between 5000 and 11,000 pseudogenes, which is at least ten times more than in pigs [46]. Vamathevan et al. described 340 pseudogenes encoded in the *de novo* assembled minipig genome [15] which is in line with the findings summarized above. Among this set of pseudogenes only 15 were described as pseudogenized drug targets including the genes for dihydrofolate reductase (DHFR), thymidylate synthetase (TYMS) and prostaglandin synthase 1 (PTGS1) also known as cyclooxygenase 1 (COX1) [15]. The products of these genes are validated targets for chemotherapy or the treatment of skin disorders. Since DHFR and TYMS are essential for *de novo* thymine nucleotide synthesis, we wished to confirm disruption of these genes together with PTGS1 in our assembly of the minipig genome. Indeed, we detected one DHFR and two TYMS pseudogenes on chromosome 1 and on chromosomes 5 and 6, respectively. In addition, we have identified functional copies encoding DHFR and PTGS1 on chromosomes 2 and 1, respectively. The predicted mRNAs encode open reading frames of the expected length and sequence identities of 99.5 % (DHFR) and 100 % (PTGS1) to the Duroc alleles (data not shown). Furthermore, we detected DHFR mRNA expression mainly in the cerebellum and PTGS1 in minipig gall bladder and lung corresponding to the pattern in human tissues (data not shown). In the Duroc pig genome, the TYMS gene could not be assigned to chromosomes but a functional allele is instead present within an unplaced scaffold assembly (NW_003539919.1). Mapping of minipig RNA sequencing reads to the Duroc TYMS template enabled reconstruction of the entire coding sequence. Furthermore, we detected transcripts in minipig intestine, spleen, liver, lung, ovary, and testis providing final proof of gene integrity (data not shown).

Using an unbiased screen across the minipig genome for pseudogenes with functional human orthologs, we have identified 12 out of 441 minipig pseudogenes with mutations affecting protein translation and integrity in both the Duroc and our minipig genome. These pseudogenes exhibit poor functional annotation with the exception of the human tumor suppressor gene HEPN1 [21, 22]. The low incidence of tumors in pigs [23–25] is consistent with a dispensable function of HEPN1 but maintenance of identical copies of this disrupted gene in African and Eurasian pig genomes is puzzling. One plausible explanation for this result would be the location of an essential gene on the opposite strand. Indeed, the last non-coding exon of the minipig ortholog of HEPACAM (also known as GlialCAM) encoding an essential human cell adhesion molecule partially overlaps the coding sequence and 3’-untranslated region of HEPN1. HEPACAM mRNA is expressed in all tissues included in this study with highest levels in brain (data not shown). In humans, the HEPACAM gene product is involved in multiple processes such as leukoencephalopathy, mental retardation, tumor suppression and leukodystrophy based on mutations associated with these disorders [47, 48].

Alternatively, HEPN1 has features of a processed pseudogene [29]. It has a single exon and we have detected significant levels of HEPN1 non-sense transcripts in all minipig tissues of this study, especially in the brain (data not shown). *Lethe* is an example of a human pseudogene that produces lncRNAs which bind the transcription factor RelA, inhibiting RelA’s ability to bind NF-kB gene promoters [49]. Transcription of both strands at the HEPN1/ HEPACAM locus combined with possible regulatory functions provides a plausible explanation for the evolutionary stability.

The availability of eukaryotic reference genomes and of four complete Eurasian pig genome sequences, plus a collection of WGS reads from the Warthog and the Bushpig combined with deep RNA sequencing libraries from liver and testis opened the possibility of identify transcripts unique to the pig family (*Suidae*). Since protein-coding gene sequence divergence between species is considered as insufficient to account for substantial lineage- and species-specific phenotypes, much more attention has been paid to regulatory sequence divergence, gene amplifications, gene loss, and lineage-specific protein-coding genes [28, 50, 51]. However all these approaches neglected the potential role of lncRNA in speciation, which are not well conserved across species and which are important regulators of gene expression [29]. In primates, for example, 131 specific lncRNAs have been identified that are absent in any known non-primate genome [28]. Using lncRNA focused filter criteria we identified 133 lncRNAs in testis tissue which were present in three independent RNA sequencing libraries and encoded in all available pig genomes with high sequence conservation in *Suidae*. The fact that this number is amazingly close to the yield in primates and that these lncRNAs are expressed in reproductive organs implicates a potential function related to reproduction. Once pluripotent pig stem cells become available, genome edited lines can be used for functional studies of these lncRNAs in cell differentiation and tissue development.

### The minipig as model for preclinical safety

The first sequence and annotation of the minipig genome combined with the Duroc farming pig genome allow protein sequence alignment with human drug targets and thereby an assessment of drug cross-reactivity. The minipig genome sequence presented in this paper further augments the reliability of genome based predictions regarding translational research. By mapping sequence alignments onto X-ray crystal structures we have shown that the PPARα contact residues for the PPARα/γ co-agonist aleglitazar are fully conserved between human and minipig, but different to rodents for three amino acids. This suggests that the receptor affinity for minipig might be close to the human value while in mouse and rat a significant loss of binding was observed. Along the same line, cross-reactivity of Avastin, a therapeutic VEGF-capturing antibody, depends on a single glycine residue that has been mutated to serine in rodents resulting in abolishment of cross-reactivity. Whether this human immune reagent shows the predicted cross-reactivity with the minipig ortholog awaits experimental verification. Quantitative tissue gene expression databases are equally important for genome based selection of a responder species. This paper closes this gap for the minipig together with the release of comprehensive tissue gene expression databases as valuable resources for the entire biomedical research community. The possibility of running an *in silico* expression analysis across 18 minipig tissues, for instance, allows the prediction of tissues where exaggerated and adverse pharmacologic effects of new drugs at their target so called on-target toxicities are possible. Furthermore knowledge about the tissue specific expression of drug metabolizing enzymes such as CYP450 isoforms or antibody activating proteases, such as MMP1, can predict the tissue specific formation of metabolites or activation of pro-drugs or pro-antibodies once the principal mechanisms of drug metabolism and elimination have been elucidated [52, 53].

Apart from applied science, we have used these databases for comparative medicine. By using a pathway centered approach and tissue-specific gene expression signatures, we have shown highly similar gene expression programs in corresponding human and minipig tissues. Especially signature genes in colon, heart, liver, and spleen have particularly high enrichment scores, suggesting similar biotransformation properties. Our minipig specific microarrays open for the first time global transcriptional profiling of drug responses or adverse effects, also referred to as toxicogenomics. Although quantitative RNA sequencing is becoming more affordable and user friendly, this technology exceeds in many cases the needs because preclinical research is commonly focused on protein targets. RNA targets, such as the lncRNAs reported here, are just emerging as therapeutic targets due to the limited understanding of their biological function [54]. In addition, data processing and analysis is straightforward and does not require complex data analysis programs or computing capacity like current sequencing technologies. An expected drawback is the low interest in minipig microarray production by commercial vendors due to the small market size as compared to human or rodent platforms. As an alternative, custom orders are affordable and efficient once the design is completed and the oligonucleotide pools are synthesized and available to the scientific community.

### Preclinical drug safety assessment in minipigs

For selection of a proper non-rodent model for drug metabolism and pharmacokinetics, the activity of the metabolizing enzymes for a given drug is a critical parameter. The elevated activity and mRNA abundance of FMO, AKR/CR and UGT in minipigs for example, are expected to affect pharmacokinetics in case one of these enzymes catalyzes conversion of a drug candidate in human liver. Thus the gene expression data that cover the entire set of drug metabolizing enzymes in minipigs and other non-rodent models are useful to guide selection of an appropriate species for pharmacodynamic and pharmacokinetic assessment of new drugs.

Following preclinical drug development, phase I clinical trials show drug safety and toxicity in human volunteers. Although data a limited, we have compared the preclinical systemic and dermal responses of seven marketed drugs targeting different disorders in minipigs and humans (Table 1). In general, the physiological responses are comparable and in case of the mTOR inhibitor Everolimus virtually identical. The availability of the minipig transcriptome allowed sequence alignment of the human and the minipig targets showing at least 89 % similarity. As a consequence, the observed similarity between the dermal and systemic responses in humans and minipigs might be related to target pharmacology.

**Table 1:** Preclinical minipig responses and clinical human responses of marketed drugs

Drug	Indication	Drug target	Gene	Minipig		Human		Interspecies target similarity
				Systemic responses	Dermal response	Systemic responses	Dermal response	
Pimecrolimus	Atopic dermatitis	Calcineurin	FKBP1A / PPP3CA	low thymus weight	none	none	mild itching	95.4 % / 94.6 %
				arteritis		-		
				lymphocytes accumulation		-		
				lung lymphoid tissue increase		-		
Everolimus	Immuno-suppression	mTOR protein kinase	FKBP1A / MTOR	Diarrhea	n.a.	Diarrhea	n.a.	95.4 % / 99.6 %
				Dermatitis		Stomatitis		
				Sedation		Sedation		
				Weak limbs		Weak limbs		
				Slow breathing		Slow breathing		
				Bloody feces		-		
Tretinoin	Acne treatment	Retinoic acid receptors	RXRB/G	n.a.	Erythema	n.a.	Erythema	98.5 % / 99.1 %
	Acute myeloid leukemia				-		Itching	
Determir insulin	Diabetes	Insulin receptor	INSR	n.a.	reversible local reactions	n.a.	reversible local reactions	~99.7 %^a^
Alendronate	Osteoporosis	farnesyl pyrophosphate synthase	FDPS	increased bone turnover in ovariectomized minipgs	n.a.	increased bone turnover in post-menopausal women	n.a.	93.8 %
Meloxicam	Rheumatic Arthritis	prostaglandin-endoperoxide synthase 2	PTGS2	Gastric ulcers	n.a.	Gastric ulcers	n.a.	>94 %^a^
				Chronic bronchopneumonia		Asthma, dyspnea, bronchospasm		
				Lethargy		Fatigue		
Carvedilol	Hyper-tension	adrenic receptor family	ADRB1/2	Cardioprotection	n.a.	Cardioprotection	n.a.	~89.3 %
			ADRA1A/1B	Reduction of infarct size		Reduction of infarct size		96.4 % / 98.7 %

^a^full minipig sequence not available

Response data were taken were taken from pre-clinical minipig drug safety toxicology studies or from public clinical trial databases. The interspecies target similarity was calculated based on protein sequence alignment

As an additional example related to the minipig as model for translational research, we compare the expression levels of genes encoding targets of marketed therapeutic antibodies covering a variety of indications including cancer or cardiovascular disorders (adapted from Waldmann et al. [55]; for details see legend to Additional file 10: Figure S5). Overall, the gene expression levels in colon, kidney, lung, heart, liver and spleen tissues are highly concordant between human and the minipig (Additional file 10: Figure S5). Since xenograft tumor models have not been established in minipigs and Cynomolgus monkeys, safety testing of tumor targeting drug candidates can only address tissue-cross-reactivity (TCR) and adverse on-target effects but not efficacy.

Since 2006, the regulatory authorities have started to request preclinical toxicology studies in juvenile animals because little is known about drug absorption, distribution, excretion or metabolism in children showing the need for translational models. The litter size and early sexual maturity are factors favoring the minipig as model for pediatric drug safety studies. In most tissues the gene expression programs are completed between two and four weeks after birth with the exception of the immune system. In contrast to humans, pigs possess an epitheliochorial placenta that is impermeable for maternal immunoglobulins. As a result, piglets are born without innate immunity and protection is achieved by delivery of protective antibodies and immune cells contained in the colostrum milk from the mother [56]. It has been shown at the cellular level, that B- and T-cell development in piglets starts four weeks after birth in the thymus and yolk sac and comparable to humans the production of immune cells shifts later in development to liver and finally to bone marrow [57]. In minipigs, we detect immune cell gene expression signatures between four and eight weeks after birth in spleen in bone marrow (Fig. 4) resembling the expression mode in humans.

Furthermore, the transcriptome analysis across safety-relevant tissues presented here opens the possibility to determine the time point during development at which expression of a particular metabolic pathway or a CYP450 variant of interest is complete. We have shown based on global transcript profiling that the developmental gene expression program is concluded about 4 weeks after birth in virtually all organs. The only study addressing juvenile toxicity in minipigs has investigated expression and activity of CYP3A4 during post-natal development [39]. Both CYP3A4 protein and enzymatic activity are detectable 28 days after birth which is in agreement with the kinetics in our dataset. Most target genes of marketed drugs expressed in adult tissues (Additional file 10: Figure S5) are detectable in equivalent juvenile tissue 4 weeks after birth (data not shown). Based on the state of executed transcriptional programs, four week old piglets should give very similar pharmacological responses as adult minipigs. Once confirmed by physiological data, this finding would significantly reduce the quantity of compounds due to the five-fold lower weight of piglets compared to adults. In addition, the entire litter and cross-fostering could be used for experiments assuring comparable genetic background thereby reducing outliers. This might be a considerable advantage because we have shown above that the number of genes with variable tissue expression is significantly higher in minipig organs than in humans (Fig. 4). The current transcriptional analysis further supports the minipig as animal model in pre-clinical research and pharmacology compliant with aims of the RETHINK project [5] and the 3R-principles for animal welfare [45]. Currently, the use of non-human primates in biomedical research is under heavy debate, and alternatives are considered especially in light of the recent advances in genetics and molecular biology [58]. Stem-cell based human models are gaining attention as in vitro models, and this study, combined with the available databases released in the public domain, will further promote the minipig as an alternative model for non-human primates such as Cynomolgus macaque *M. fascicularis*. Finally, pigs offer the unique opportunity of generation large animal models for human disease using nuclear transfer from custom engineered fibroblast cells. Current porcine models for human disease include various cancers, cystic fibrosis, Duchenne muscular dystrophy, autosomal polycystic kidney disease, Huntington’s disease and spinal muscular atrophy [1].

## Methods

### Minipig tissue samples

Approval by an ethics committee was not required for this study because all minipig tissue samples were obtained as catalogue item from Ellegaard Göttingen Minipigs A/S, Dalmose, Denmark (http://minipigs.dk/ordering/). All tissues came from six naïve female and 6 male in accordance with current animal welfare standards (http://minipigs.dk/the-goettingen-minipig/animal-welfare/). Details (gender, weight, age, family relationship) of all animals are on record and are included in our microarray data submission to GEOS.

### Minipig genome sequencing

Genomic DNA was isolated using the QIAamp DNA Mini Kit (Qiagen Inc., Valencia, CA, USA) from liver tissue of a 1 year old female Göttingen minipig from Ellegaard. For Roche-454-sequencing 1 μg of DNA was mechanically sheared to an average length of 320 bases and processed with the GS FLX Titanium Rapid Library preparation kit. In addition, Roche-454 paired-end libraries with two end tags of ~140 base-pairs (bp), separated by an eight kilobase (kb) insert, were generated from 15 μg DNA with the GS FLX Titanium Paired End Adaptor sets to improve unique read alignments and sequence gap-filling (Roche-454, Brandford, CT, USA). For SOLiD-sequencing of 50 bp single-end reads, single fragment libraries were generated from 5 μg sheared DNA with the fragment library core kit. 5 μg of DNA were used for the construction of a paired-end fragment library with 50 bp forward and 25 bp reverse reads and 1–3 kb insert size using the mate-paired library kit (ABI/ LifeTechnologies, Carlsbad, CA, USA). All libraries were amplified by emulsion PCR prior to sequencing. Thirty-two Roche-454-FLX single-read and two paired-end read runs were performed and complemented by three SOLiD-3-plus single-end read runs and one paired–end read sequencing run. Roche-454 long reads and SOLiD short reads were mapped and assembled to the Duroc reference genome (Sscrofa 10.2) by using a template-based approach as described previously [41]. Only reads mapping uniquely to the template reference genome were incorporated into the minipig genome assembly. In addition, Roche-454 reads that could not have been anchored to chromosomes of the minipig genome were assembled de-novo with the Roche-454 Newbler software (version 2.5.3).

### Porcine specific transcripts

Normalized Roche-454 SAGE (serial analysis of gene expression) libraries of RNA reads were combined with paired-end Illumina Genome Analyser RNA reads and assembled in 3’686 contigs longer than 500 nucleotides using Roche-454 Newbler (version 2.5.3) and Trinity [59] software packages with default algorithm parameters, respectively (Additional file 7: Figure S3). By applying BLAST [16], the resulting contig pool was mapped on the predicted minipig mRNAs, RefSeq pig mRNAs and Swiss-Prot in order to remove protein-coding transcripts. Next, we applied Genomic Mapping and Alignment Program (GMAP) [60] to deplete contigs matching other vertebrate genomes: a total of 16 genomes (cow, horse, dog, human, orang utan, chimpanzee, cynomolgus monkey, rhesus monkey, marmoset, rabbit, guinea pig, hamster, mouse, rat, opossum, chicken) was utilized. This workflow generated a set of 133 pig-specific long-non-coding RNAs, which exhibited significant expression above 10 RPKM (reads per kilobase of transcript per million reads mapped) as determined by Illumina paired-end sequencing and SOLiD Serial Analysis of Gene Expression (SAGE).

### African pig genome sequencing

Genomic DNA was isolated using the QIAamp DNA Mini Kit (Qiagen Inc., Valencia, CA, USA) from blood samples of an African Warthog (*Phacochoerus africanus*) and two Bushpigs (*Potamochoerus larvatus)* obtained from the International Livestock Research Institute, Kenya (ILRI). 1 μg DNA was used as input for the Ion Xpress Plus genomic DNA fragment library preparation kit for whole-genome shotgun sequencing. After emulsion PCR, libraries with a median size of ~250 bp were subjected to semiconductor sequencing using the Ion Proton system (LifeTechnologies, Carlsbad, CA, USA). Reads were mapped with GMAP (http://www.gmaptool.eu/en) to the minipig genome draft as described above.

### RNA sequencing

Total RNA was extracted from minipig testis using the RNeasy Mini kit combined with DNase treatment on a solid support (Qiagen Inc., Valencia, CA, USA). For unstranded RNA sequencing, 4 μg total RNA were either treated with oligo(dT)_25_ Dynabeads to enrich poly-A RNA or with the Ribo-Zero Magnetic Gold kit for hybridization dependent depletion of ribosomal RNA (Epicentre, Madison, WI, USA). Poly-A selected and ribosomal depleted RNA was used for Illumina library generation with the ScriptSeq v2 RNA-seq kit. 100 bp paired-end sequencing was performed on an Illumina Genome Analyzer II_X_ . For Roche-454 sequencing, poly-A selected and ribosomal depleted RNA was used for random primed cDNA (complementary DNA) library generation with the Roche cDNA-synthesis system in combination with the GS FLX Titanium Rapid Library preparation kit. The SOLiD SAGE kit was used to generate a library of 27 bp tags per transcript with from poly-A enriched and ribosomal depleted RNA (ABI/LifeTechnologies, Carlsbad, CA, USA). After emulsion PCR, Roche-454 and SOLiD libraries were sequenced as single-end read runs on a Roche-454 FLX or the SOLiD-3-plus sequencing system. Roche-454 and SOLiD SAGE reads were combined with paired-end Illumina reads to assemble and quantify contigs with longer than 500 base pairs using Roche-454 Newbler (version 2.5.3) or Trinity assembler software with default algorithm parameters, respectively [59].

### Design of minipig specific microarrays

We have used our predicted minipig transcriptome complemented by non-overlapping pig mRNA sequences from the RefSeq database for custom microarray design to monitor the expression of ~17,000 genes. For this type of microarray, 60-mer oligonucleotide hybridization probes were manufactured either with NimbleGen photolithography or with Agilent inkjet printing technology after closure of Nimblegen’s microarray business segment. Using technical replicates and ERCC (external RNA controls consortium) spike in controls, these microarrays exhibited a high reproducibility within replicates (mean r^2^ = 0.994; *n* =12) and an average dynamic range of 8 log_2_ units (*n* = 12). The lower limit of detection was determined with ERCCs at the signal level of random probes, which serve as a metric of non-specific annealing and background fluorescence. Furthermore we used SOLiD and Roche-454 RNA-sequencing data from four minipig tissues (liver, spleen, heart, kidney) to proof that the *in silico* designed probes matched to experimentally determined mRNA sequences. As it turned out, the full-length sequence of ~80 % of all probes was present in the transcript pool, ~10 % of the probes had imperfect homology, and ~10 % of all probes had no match to the RNA-sequencing data.

### Microarray-based gene expression analysis

Minipig tissues were homogenized in tubes prefilled with 1.4 mm ceramic beads and QiaGen’s lysis buffer using a FastPrep-24 instrument (MP Biomedicals, Solon, OH, USA). Total RNA from lysates was extracted using the RNeasy Mini kit combined with DNase treatment on a solid support (Qiagen Inc., Valencia, CA, USA). RNA quality assessment and quantification was performed using microfluidic chip analysis on an Agilent 2100 bioanalyzer (Agilent Technologies Inc., Santa Clara, CA, USA). For NimbleGen microarrays, 10 ng of total RNA was used to prepare cDNA on a Biomek FXp workstation (Beckman Coulter Inc., Brea, CA, USA) with the NuGen Ovation Pico WTA System V2 (NuGEN Technologies, Inc., SanCarlos, CA, USA), followed by Cy3 labeling of cDNA with the Roche NimbleGen One Color DNA Labeling Kit. NimbleGen 12x135K gene expression microarrays (Design-ID 120229_MiniPig_TH_expr_HX12) were hybridized with 4 μg of Cy3-labeled cDNA (copyDNA) for 16 h at 42 °C. For Agilent microarrays, 100 ng of total RNA was used to prepare Cy3-labled cRNA (copyRNA) using the Agilent Low Input Quick Amp Labeling kit (Agilent Technologies Inc., Santa Clara, CA, USA), followed by QiaGen RNeasy column purification. 600 ng Cy3-labelled cRNA was fragmented at 60 °C for 30 min and hybridized to Agilent SurePrint G3 Custom GE Arrays 8x60K (Design-ID 050244) for 17 h at 65 °C in a rotating Agilent hybridization oven. After hybridization, NimbleGen and Agilent microarrays were washed and dried according to the manufacturer’s instruction. Microarray data was collected by confocal scanning using the Roche NimbleGen MS200 Microarray Scanner at 2 μm pixel resolution (Roche NimbleGen, Inc., Madison, WI, USA). Probe intensities were subjected to Robust Multi-Array Analysis (RMA) with background correction and quantile normalization. Averaged gene-level signal intensities were summarized into gene calls and log_2_ transformed. Data analysis and visualization was performed using Partek Genomics Suite version 6.6 (Partek, Inc., St. Louis, MI, USA), Spotfire version 6.5.2 (Tibco Software Inc, Boston, MA, USA), and the R software for statistical computing and graphics (R-Development-Core-Team 2008. R: A language and environment for statistical computing. R Foundation for Statistical Computing, Vienna, Austria). Enrichment analysis of Gene Ontology (GO) annotations was performed using the DAVID Bioinformatics Resource (https://david.ncifcrf.gov/).

### Definition of human tissue gene expression signatures

Tissue-specific gene signatures were identified from three datasets: NB [30] and GNF [61], both based on Affymetrix microarrays, and GTEx [62]. Gini index [63] was used to identify tissue-specific genes. The calculation of the Gini index was performed in “R” using the “ineq” package. The Gini index ranges between zero (gene is ubiquitously and uniformly present or abenst in all tissues) and one (gene is exclusively expressed in one tissue). We define a gene as a tissue signature gene if the Gini index equals 0.7 or greater.

### Human reference microarray data analysis

For comparison of minipig with human mRNA expression values we used a public data set from NCBI Gene Expression Omnibus (accession no. GSE3526). Human tissues from 3 to 9 different adult Caucasian donors were analyzed on Affymetrix HG-U133-PLUS_2 chips. For inter-species comparisons expression data from 16,032 common genes and 13 common tissues was normalized using the Global Rank-invariant Set Normalization (GRSN) to reduce systematic distortions in microarray data [31].

### Metabolic activity measurements

Hepatocyte suspension cultures were prepared from commercial cryopreserved hepatocytes from non-transplantable liver tissues purchased from Promocell (Vitaris AG, 6340 Baar, Switzerland). For minipig, commercially available cryopreserved male Göttingen minipig hepatocytes were purchased (BioreclamationIVT, USA, Product Number M00615, lot Number XNG). Suspension cultures were grown with gentle shaking in William’s media supplemented with glutamine, antibiotics, insulin, dexamethasone and 10 % fetal calf serum (FCS). The final concentration of the reporter substrates midazolam, dextromethorphan, diclofenac, tolbutamide, bupropion and 7-hydroxycoumarin was 1 μmolar. O6-benzyl guanine, tacrine, SN-38 and sulfamethazine were incubated from 2.5, 10, 50 to 50 μmolar. Hepatocytes were grown in 96-well suspension cultures (1 million cells/ml) in plates shaking (900 revolutions per minute) for 2 h in a 5 % CO_2_ atmosphere at 37 °C. At defined time points, 100 μl of the cell suspension in each well was quenched with 200 μl acetonitrile containing an internal standard. Samples were then cooled and centrifuged followed by quanitification by using liquid chromatography and mass spectrometry (LC-MS/MS). The parameter settings for LC/MS analyte detection in positive or negative ion MRM mode are summarized in Additional file 11: Table S6. The Shimadzu HPLC (high pressure liquid chromatography) system consisted of 10ADvp pumps connected to a 5000 AB Sciex mass spectrometer equipped with a TurboIon-Spray source (IonSpray Voltage 4500V in negative mode) and a HTS CTC PAL autosampler. For 1-OH midazolam, benzydamine N-oxide, daunorubicinol, N-acetyl-sulfamethazine, SN-38 glucuronide, 8-oxo-O6-benzylguanine, hydroxybupropion, 4-hydroxydiclofenac, dextrorphan and hydroxytacrine, a 50 × 2 mm analytical column with 5 μm particle size Phenomenex Gemini C18 110A resin was used. 7-hydroxycoumarin glucuronide and sulfate, were chromatographed using a 50 cm × 2 mm column with Phenomenex, Synergi Hydro-RP 80 Å resin with 4 μm particle size at 40 °C. Mobile phase A was 0.2 % formic acid in water, mobile phase B was 0.1 % formic acid in water/methanol 95:5. 1 μl aliquots of the centrifuged sample solutions were injected and transferred onto the analytical column at a flow rate of 0.50 mL/min using 95 % mobile phase A. To elute the compounds, a high pressure, linear gradient from 5 to 95 % B in 98 s was applied. A minimum of 6 calibration standards with a precision of 20 % and accuracy between 80 and 120 % were used for calibration. Data analysis was performed using weighted (1/x2) linear regression on analyte/internal standard area ratios. For data analysis, Analyst 1.4.2 software was used. To derive metabolic rates, the determined concentrations of the metabolites were plotted against time and a linear fit made to the data with emphasis upon the initial linear rate. The initial linear rate was then used to derive at the metabolite formation rate (pmol/min/million cells).

### Availability of supporting data

The minipig whole genome shotgun project has been deposited at DDBJ/EMBL/GenBank under the accession LIDP00000000. The microarray data from this study have been deposited at the NCBI Gene Expression Omnibus (GEO) (http://www.ncbi.nlm.nih.gov/geo/) under accession numbers GSE71438 and GSE71441. PDB code for crystal structures (http://www.rcsb.org/): 3G8I, 1FLT.

## Additional files

Additional file 1: Table S1. Minipig genome assembly statistics. Only reads mapping uniquely to the Duroc sus scrofa reference genome were incorporated into the minipig genome assembly. (DOCX 13 kb) Additional file 2: Table S2. Contig assembly statistics. Roche-454 reads that were not incorporated into the minipig genome were assembled de-novo with Roche-Newbler software. (DOCX 13 kb) Additional file 3: Table S3. Mapping rates of Duroc gene sequences to available porcine genomes. (DOCX 13 kb) Additional file 4: Figure S1. Sequence comparisons across different pig species. 20’786 gene sequences from the Duroc pig genome *Sus Scrofa* 10.2 as from ENSEMBL were mapped onto the genomes of Roche minipig, the *de novo* assembled Göttingen minipig from Vamathevan et al. and the Tibetan Pig v1.0 from Novogene using Blast. For each of the 3 pig genomes, the relative number of the orthologous gene sequences was plotted against the sequence identities to the Duroc pig genes. (JPEG 606 kb) Additional file 5: Table S4. Disrupted minipig orthologs of human protein coding genes. (DOCX 14 kb) Additional file 6: Figure S2. Sequence analysis of the HEPN1 pseudogene. Alignment of the human HEPN1 mRNA sequence (NM_001037558) with orthologs from the minipig, the Duroc Pig, *Phacochoerus africanus* (Warthog) and *Potamochoerus larvatus* (Bushpig. The A/G start-codon mutation is highlighted in green, insertions or deletions are in yellow, stop-codons in red and conserved sequences are colored in magenta. (PDF 115 kb) Additional file 7: Figure S3. Schematic description of the *in silico* workflow for selection of *Sus scrofa* specific lncRNAs. * 16 genomes: cow, horse, dog, human, orang utan, chimpanzee, cynomolgus monkey, rhesus monkey, marmoset, rabbit, guinea pig, hamster, mouse, rat, opossum, chicken. See text for more detailed description. (JPEG 854 kb) Additional file 8: Figure S4. Sequence alignment of 133 minipig lncRNAs with genomic copies in the Roche minipig, the *de novo* assembled minipig from Vamathevan et al., the Tibetan boar and the Duroc pig. The color scale indicates the alignment coverage or presence of the lncRNAs within the corresponding genomes. 100 % presence and absence are denoted by black and yellow, respectively. The red box zooms into regions with incomplete coverage. Parts of the mismatches are likely due to misassembled loci (see text for details). (JPEG 1544 kb) Additional file 9: Table S5. Mapping rates of Bushpig and Warthog WGS reads to minipig lncRNA sequences. (DOCX 28 kb) Additional file 10: Figure S5. mRNA expression levels of target genes of marketed therapeutic antibodies in human and minipig tissues. Radar chart (log scale) plotting shows expression levels of human drug targets starting clockwise at the green arrow on top clockwise. Expression levels of the following genes are displayed: ADRA1A, ADRA1B, ADRB1, ADRB2, C5, CD33, CD3D, CTLA4, EGFR, ERBB2, FDPS, IL1B, IL2RA, IL6R, INSR, ITGA4, ITGAL, MS4A1, MTOR, PPP3CA, PTGS1, PTGS2, RXRB, RXRG, TNF, TNFRSF8, TNFSF13B, VEGFA. The blue line shows normalized human expression levels (log2 levels), and the red line depicts equivalent minipig data in each tissue. The dotted black circle marks the adapted detection limit of the microarray platforms used. The circular grey background scale shows the scale in two log2 intervals. (JPEG 3537 kb) Additional file 11: Table S6. Parameter settings for LC/MS analyte detection in positive or negative ion MRM mode. (DOCX 14 kb)
