# Supplementary material for: Functional analysis and transcriptional output of the Göttingen minipig genome
Source: BMC Genomics. 2015 Nov 14;16:932. doi: 10.1186/s12864-015-2119-7 (PMC4647470; doi:10.1186/s12864-015-2119-7)
Supplement: Additional file 1: Table S1. — Minipig genome assembly statistics. Only reads mapping uniquely to the Duroc sus scrofa reference genome were incorporated into the minipig genome assembly. (DOCX 13 kb) [file 12864_2015_2119_MOESM1_ESM.docx]

**Additional file 1: Table S1** Minipig genome assembly statistics.

Only reads mapping uniquely to the Duroc sus scrofa reference genome were incorporated into the minipig genome assembly.

Average coverage 20.8 fold

Number of chromosomes 18 plus Chr. X

Total length of genome 2.60 Gb (including 0.6 Gb of N’s^2^)

Number of assembled Roche-454 reads^1^ 28 M (8.9 Gb)

Number of assembled single-end SOLiD reads 550 M (27 Gb)

Number of assembled paired-end SOLiD reads 243 M (21 Gb)

^1^ single- and paired-end reads

^2^ Gb; gigabase, N's; G, A, T or C
