# Supplementary material for: Functional analysis and transcriptional output of the Göttingen minipig genome
Source: BMC Genomics. 2015 Nov 14;16:932. doi: 10.1186/s12864-015-2119-7 (PMC4647470; doi:10.1186/s12864-015-2119-7)
Supplement: Additional file 2: Table S2. — Contig assembly statistics. Roche-454 reads that were not incorporated into the minipig genome were assembled de-novo with Roche-Newbler software. (DOCX 13 kb) [file 12864_2015_2119_MOESM2_ESM.docx]

**Additional file 2: Table S2:** Contig assembly statistics

Roche-454 reads that were not incorporated into the minipig genome were assembled de-novo with Roche-Newbler software.

Input reads^1^ 2.9 M (0.97 Gb)

N50 contig size 946

N50 contig number 39’130

N50 scaffold size 3’356

N50 scaffold number 1’926

Total contig size (kb) 106’280

Total contig number (≥ 500 bp) 116’078

Total scaffold size (kb) 16’701

Total scaffold number (≥ 1’500 bp) 5’187

Scaffolds placed on chromosomes^2^ n/a

^1^ single- and paired-end reads

^2^ not applicable because only reads that cannot be mapped to the genome are used
