# Supplementary material for: Functional analysis and transcriptional output of the Göttingen minipig genome
Source: BMC Genomics. 2015 Nov 14;16:932. doi: 10.1186/s12864-015-2119-7 (PMC4647470; doi:10.1186/s12864-015-2119-7)
Supplement: Additional file 3: Table S3. — Mapping rates of Duroc gene sequences to available porcine genomes. (DOCX 13 kb) [file 12864_2015_2119_MOESM3_ESM.docx]

**Additional file 3: Table S3:** Gene mapping rates

20’786 gene sequences from the Duroc pig genome SusScrofa 10.2 as from ENSEMBL were mapped on the 4 genome drafts using BLAST.

| genomes | mapped pig genes | human orthologs | pseudogenes |
| --- | --- | --- | --- |
| Sus Scrofa 10.2 | 20’786 (100%) | 19’720 (94.9%) | 457 (2.2%) |
| minipig, Roche | 20’197 (97.2%) | 19’228 (92.5%) | 441 (2.1%) |
| minipig, *de novo assembled* | 20’669 (99.4%) | 19’644 (94.5%) | 449 (2.2%) |
| Tibetan Pig v1.0, Novogene | 20’737 (99.8%) | 19’665 (94.6%) | 454 (2.2%) |
