# Supplementary figures and images for: Functional analysis and transcriptional output of the Göttingen minipig genome
Source: BMC Genomics. 2015 Nov 14;16:932. doi: 10.1186/s12864-015-2119-7 (PMC4647470; doi:10.1186/s12864-015-2119-7)

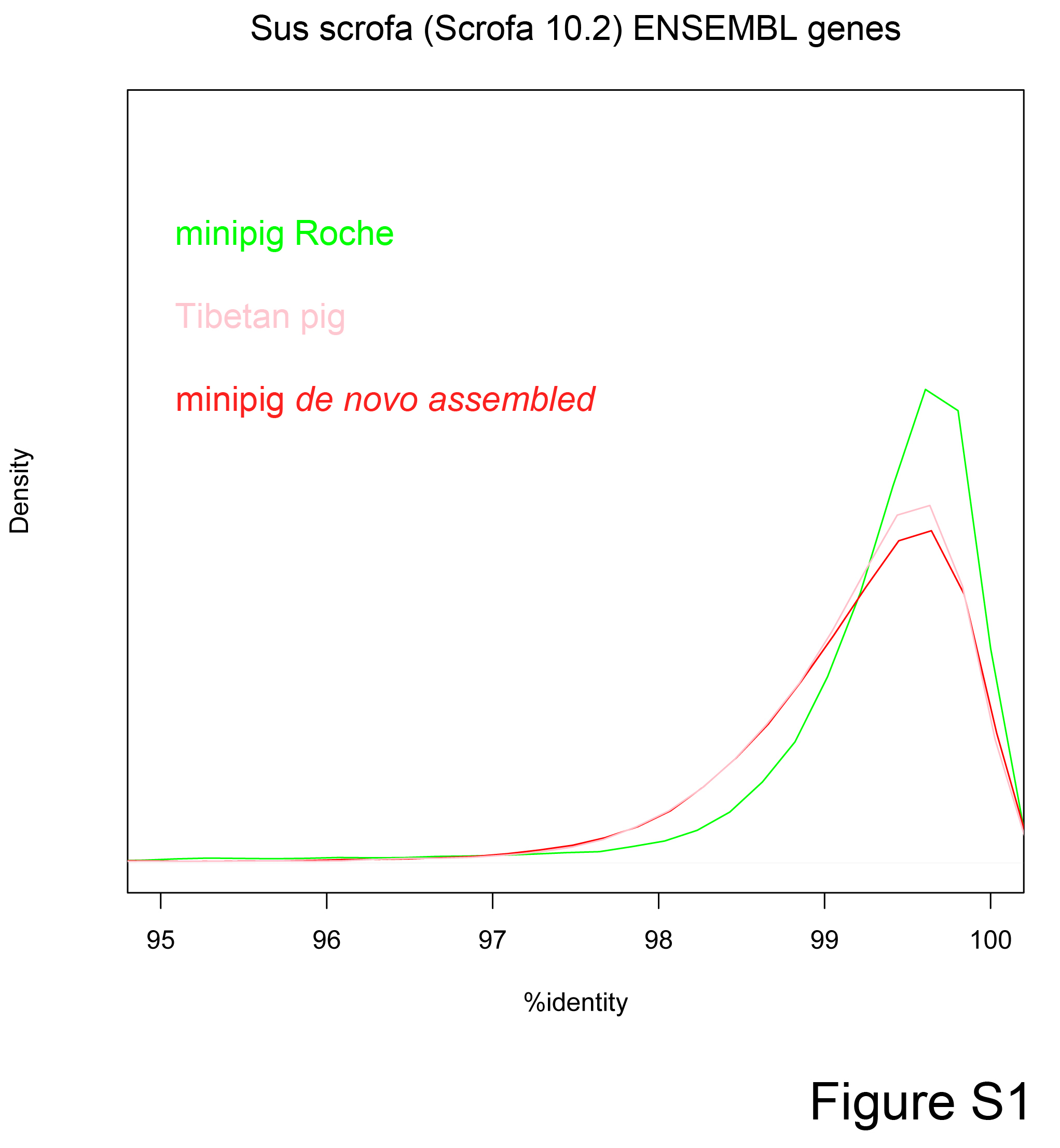

Supplement: Additional file 4: Figure S1. — Sequence comparisons across different pig species. 20’786 gene sequences from the Duroc pig genome Sus Scrofa 10.2 as from ENSEMBL were mapped onto the genomes of Roche minipig, the de novo assembled Göttingen minipig from Vamathevan et al. and the Tibetan Pig v1.0 from Novogene using Blast. For each of the 3 pig genomes, the relative number of the orthologous gene sequences was plotted against the sequence identities to the Duroc pig genes. (JPEG 606 kb) [file 12864_2015_2119_MOESM4_ESM.jpg]

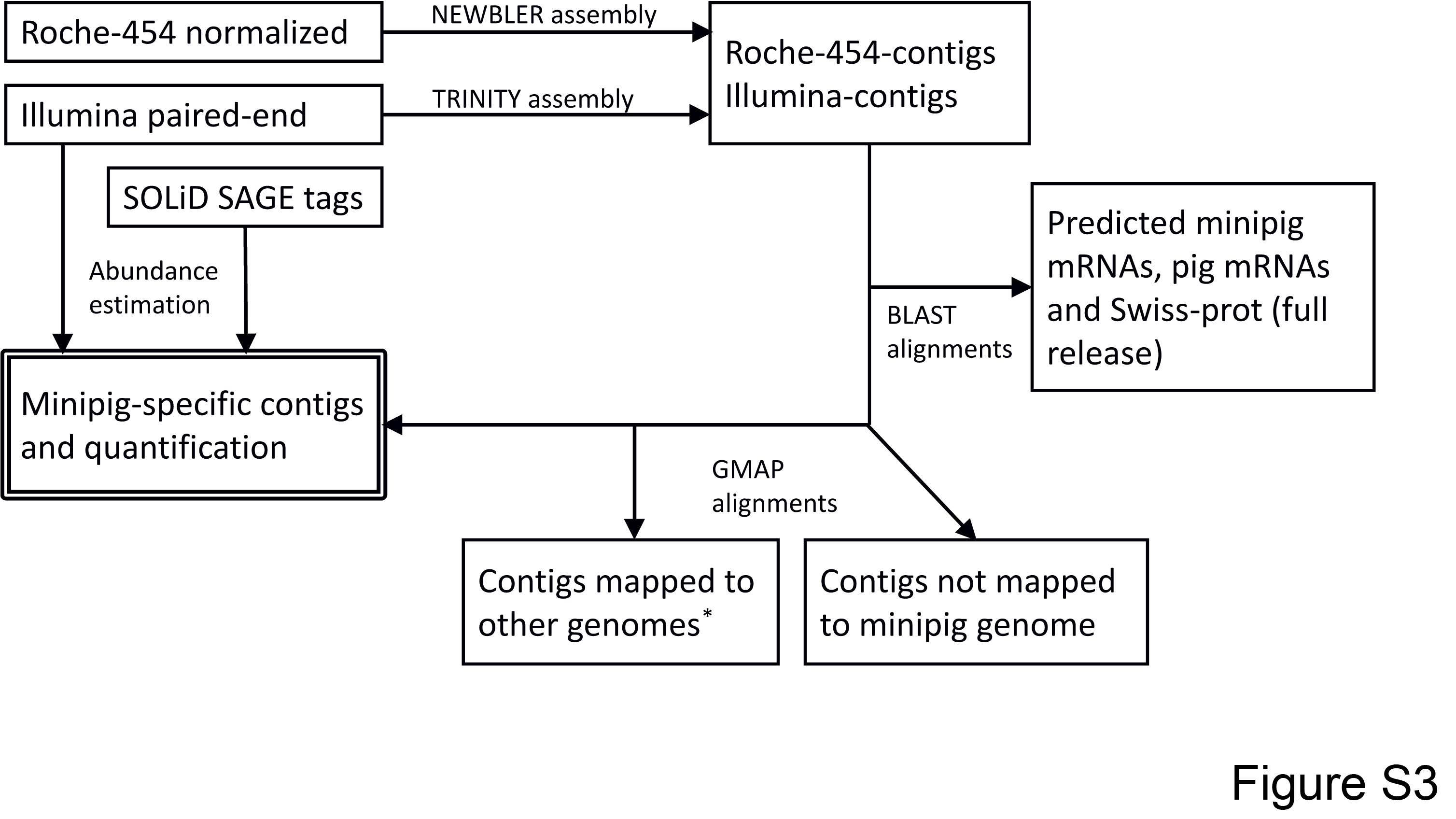

Supplement: Additional file 7: Figure S3. — Schematic description of the in silico workflow for selection of Sus scrofa specific lncRNAs. * 16 genomes: cow, horse, dog, human, orang utan, chimpanzee, cynomolgus monkey, rhesus monkey, marmoset, rabbit, guinea pig, hamster, mouse, rat, opossum, chicken. See text for more detailed description. (JPEG 854 kb) [file 12864_2015_2119_MOESM7_ESM.jpg]

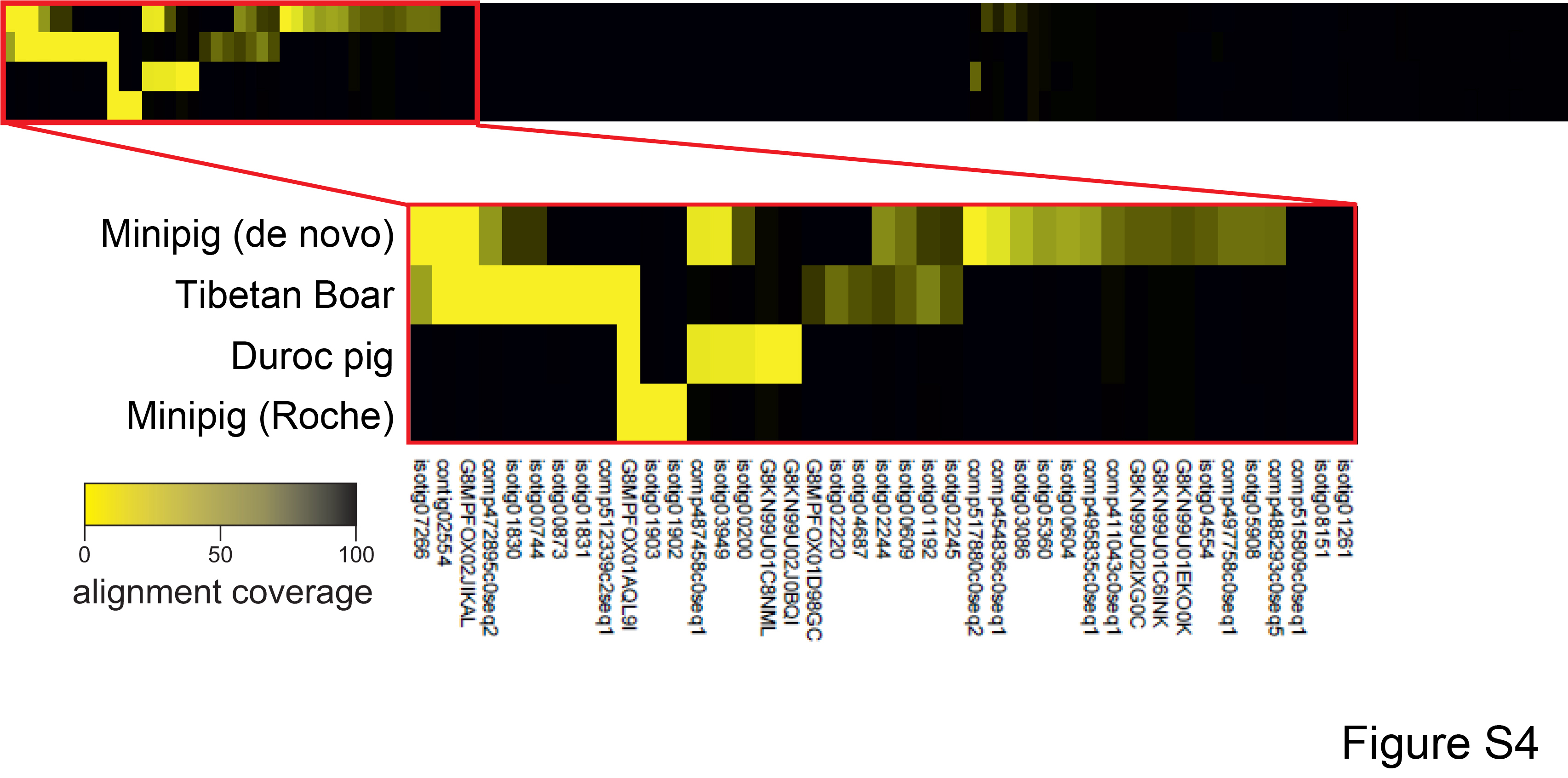

Supplement: Additional file 8: Figure S4. — Sequence alignment of 133 minipig lncRNAs with genomic copies in the Roche minipig, the de novo assembled minipig from Vamathevan et al., the Tibetan boar and the Duroc pig. The color scale indicates the alignment coverage or presence of the lncRNAs within the corresponding genomes. 100 % presence and absence are denoted by black and yellow, respectively. The red box zooms into regions with incomplete coverage. Parts of the mismatches are likely due to misassembled loci (see text for details). (JPEG 1544 kb) [file 12864_2015_2119_MOESM8_ESM.jpg]

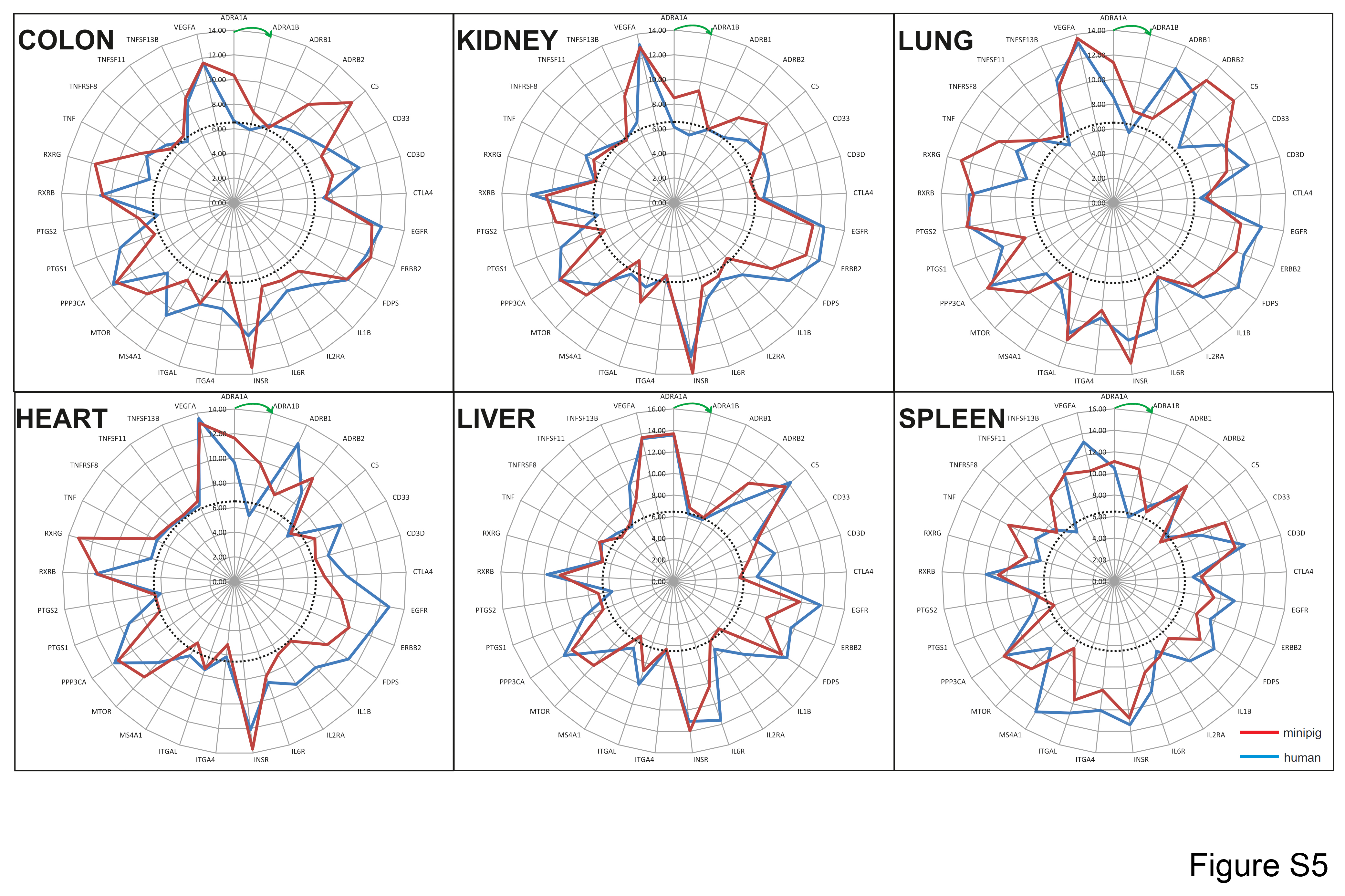

Supplement: Additional file 10: Figure S5. — mRNA expression levels of target genes of marketed therapeutic antibodies in human and minipig tissues. Radar chart (log scale) plotting shows expression levels of human drug targets starting clockwise at the green arrow on top clockwise. Expression levels of the following genes are displayed: ADRA1A, ADRA1B, ADRB1, ADRB2, C5, CD33, CD3D, CTLA4, EGFR, ERBB2, FDPS, IL1B, IL2RA, IL6R, INSR, ITGA4, ITGAL, MS4A1, MTOR, PPP3CA, PTGS1, PTGS2, RXRB, RXRG, TNF, TNFRSF8, TNFSF13B, VEGFA. The blue line shows normalized human expression levels (log2 levels), and the red line depicts equivalent minipig data in each tissue. The dotted black circle marks the adapted detection limit of the microarray platforms used. The circular grey background scale shows the scale in two log2 intervals. (JPEG 3537 kb) [file 12864_2015_2119_MOESM10_ESM.jpg]
