# Supplementary material for: Functional analysis and transcriptional output of the Göttingen minipig genome
Source: BMC Genomics. 2015 Nov 14;16:932. doi: 10.1186/s12864-015-2119-7 (PMC4647470; doi:10.1186/s12864-015-2119-7)
Supplement: Additional file 5: Table S4. — Disrupted minipig orthologs of human protein coding genes. (DOCX 14 kb) [file 12864_2015_2119_MOESM5_ESM.docx]

**Additional file 5: Table S4**: Disrupted minipig orthologs of human protein coding genes.

Genes whose coding region, as opposed to human, is interrupted by mutations present in different porcine genomes.

| **Gene symbol** | **Gene ID** | **Gene Description** | **change** |
| --- | --- | --- | --- |
| KLLN | 100144748 | killin, p53-regulated DNA replication inhibitor | frame shift |
| TRPC5OS | 100329135 | TRPC5 opposite strand | stop codon |
| SPHAR | 10638 | S-phase response (cyclin related) | frame shift |
| DEFB107A | 245910 | defensin, beta 107A | frame shift |
| KAAG1 | 353219 | kidney associated antigen 1 | frame shift |
| C15orf54 | 400360 | chromosome 15 open reading frame 54 | INDEL |
| C10orf55 | 414236 | chromosome 10 open reading frame 55 | INDEL |
| DEFB107B | 503614 | defensin, beta 107B | INDEL |
| DCAF16 | 54876 | DDB1 and CUL4 associated factor 16 | stop codon |
| HEPN1 | 641654 | hepatocellular carcinoma, down-regulated 1 | INDEL |
| C10orf95 | 79946 | chromosome 10 open reading frame 95 | INDEL |
| FANCF | 2188 | Fanconi anemia, complementation group F | INDEL |
