# Supplementary material for: Functional analysis and transcriptional output of the Göttingen minipig genome
Source: BMC Genomics. 2015 Nov 14;16:932. doi: 10.1186/s12864-015-2119-7 (PMC4647470; doi:10.1186/s12864-015-2119-7)
Supplement: Additional file 9: Table S5. — Mapping rates of Bushpig and Warthog WGS reads to minipig lncRNA sequences. (DOCX 28 kb) [file 12864_2015_2119_MOESM9_ESM.docx]

**Additional file 9: Table S5**: WGS reads of pooled Bushpig and Warthog libraries (animals BP04, BP05, WT07, and WT27) were mapped to the assembled minipig lncRNA contig sequences. The total number of reads and the coverage percentage to individual lncRNA is indicated. lncRNAs detected in one pig species or absent in both are color coded as indicated in the legend at the bottom of the table.

|  |  | **Bushpig** | | **Warthog** | |
| --- | --- | --- | --- | --- | --- |
| **ncRNA identifier** | **Length of ncRNA** | **Number of reads mapped to ncRNA** | **ncRNA covered with reads in % of length** | **Number of reads mapped to ncRNA** | **ncRNA covered with reads in % of length** |
| isotig03406 | 1'612 | 119 | 97.0% | 270 | 100.0% |
| isotig01912 | 1'093 | 31 | 88.0% | 22 | 81.0% |
| comp495835c0seq1 | 1'019 | 4 | 31.0% | 2 | 17.0% |
| isotig03848 | 1'003 | 6 | 68.0% | 4 | 58.0% |
| isotig03949 | 964 | 2 | 37.0% | 6 | 41.0% |
| comp515809c0seq1 | 920 | 9 | 64.0% | 4 | 51.0% |
| isotig04123 | 907 | 5 | 55.0% | 13 | 91.0% |
| isotig04211 | 890 | 13 | 73.0% | 20 | 96.0% |
| isotig00744 | 865 | 757 | 100.0% | 321 | 98.0% |
| G8KN99U02H2SIJ | 861 | 5 | 36.0% | 2 | 23.0% |
| isotig01830 | 856 | 157 | 98.0% | 79 | 62.0% |
| isotig01902 | 832 | 5 | 53.0% | 8 | 77.0% |
| comp517880c0seq2 | 822 | - | 0.0% | 1 | 17.0% |
| isotig04554 | 817 | 38 | 92.0% | 36 | 95.0% |
| isotig00604 | 809 | 2 | 39.0% | - | 0.0% |
| isotig04687 | 809 | 37 | 85.0% | 16 | 79.0% |
| G8KN99U01DQL53 | 808 | 9 | 83.0% | 6 | 43.0% |
| isotig01831 | 807 | 1'055 | 100.0% | 432 | 100.0% |
| isotig04738 | 799 | 8 | 66.0% | 4 | 54.0% |
| isotig04781 | 796 | 2 | 42.0% | 4 | 20.0% |
| contig01655 | 789 | 5 | 59.0% | 4 | 74.0% |
| contig02554 | 787 | 47 | 99.0% | 18 | 75.0% |
| isotig00200 | 787 | 2 | 25.0% | 1 | 16.0% |
| isotig04854 | 783 | 3 | 43.0% | 6 | 70.0% |
| isotig02012 | 779 | 2 | 31.0% | 4 | 38.0% |
| isotig02140 | 769 | 1 | 18.0% | 14 | 65.0% |
| isotig00647 | 761 | 828 | 100.0% | 384 | 100.0% |
| isotig01192 | 760 | 6 | 44.0% | 6 | 37.0% |
| comp512339c2seq1 | 759 | 3 | 49.0% | 8 | 75.0% |
| isotig05076 | 757 | 5 | 43.0% | 5 | 52.0% |
| comp502823c0seq1 | 744 | 7 | 67.0% | 6 | 43.0% |
| G8KN99U02J0BQI | 736 | 2 | 25.0% | 5 | 43.0% |
| G8MPFOX02G92EC | 736 | 2 | 35.0% | 5 | 29.0% |
| isotig00212 | 736 | 2 | 25.0% | 7 | 80.0% |
| isotig01903 | 732 | 6 | 65.0% | 7 | 82.0% |
| comp497758c0seq1 | 728 | 3 | 49.0% | 5 | 49.0% |
| isotig05360 | 727 | 5 | 44.0% | 7 | 74.0% |
| G8KN99U02GFCPG | 713 | 4 | 31.0% | 3 | 48.0% |
| comp509750c3seq1 | 711 | 3 | 34.0% | 5 | 51.0% |
| comp514119c2seq1 | 708 | 4 | 61.0% | 9 | 75.0% |
| G8KN99U01EO01T | 702 | 3 | 39.0% | 3 | 25.0% |
| G8KN99U02IXG0C | 700 | 3 | 42.0% | 8 | 74.0% |
| comp518427c0seq1 | 694 | - | 0.0% | - | 0.0% |
| isotig02244 | 692 | - | 0.0% | - | 0.0% |
| G8KN99U01C8NML | 689 | 6 | 70.0% | 4 | 59.0% |
| isotig01261 | 687 | - | 0.0% | 1 | 13.0% |
| G8KN99U02JZLC9 | 684 | 2 | 46.0% | 3 | 43.0% |
| isotig05908 | 676 | 1 | 22.0% | 5 | 44.0% |
| G8KN99U01C5O7D | 671 | 2 | 16.0% | 1 | 10.0% |
| comp492591c3seq1 | 663 | 5 | 41.0% | 15 | 96.0% |
| isotig02220 | 661 | 4 | 56.0% | 2 | 31.0% |
| G8KN99U02GGCGD | 659 | 7 | 51.0% | 4 | 54.0% |
| comp472895c0seq2 | 656 | 15 | 89.0% | 1 | 22.0% |
| G8KN99U02HKB7K | 652 | 1 | 9.0% | - | 0.0% |
| G8KN99U02G347T | 647 | 1 | 25.0% | 4 | 41.0% |
| isotig06347 | 647 | 5 | 74.0% | 12 | 87.0% |
| isotig06384 | 645 | 2 | 16.0% | 4 | 43.0% |
| isotig06470 | 641 | 6 | 62.0% | 4 | 58.0% |
| comp454836c0seq1 | 640 | 2 | 29.0% | 11 | 75.0% |
| G8KN99U02IJA84 | 639 | 4 | 65.0% | 1 | 23.0% |
| G8KN99U01A7YS1 | 636 | 1 | 26.0% | 1 | 24.0% |
| G8MPFOX02JC4MR | 635 | 23 | 95.0% | 35 | 54.0% |
| contig01609 | 629 | - | 0.0% | 8 | 66.0% |
| isotig01392 | 628 | 2 | 45.0% | - | 0.0% |
| isotig06763 | 625 | 3 | 43.0% | 4 | 62.0% |
| comp491681c1seq1 | 624 | 2 | 43.0% | 2 | 28.0% |
| isotig00873 | 618 | 7 | 83.0% | 2 | 38.0% |
| G8MPFOX02GQRJS | 616 | 242 | 100.0% | 74 | 99.0% |
| isotig06897 | 616 | 2 | 38.0% | 7 | 50.0% |
| G8MPFOX02GWZFV | 615 | 3 | 51.0% | 2 | 38.0% |
| comp518818c1seq12 | 613 | 2 | 36.0% | 4 | 51.0% |
| G8KN99U02GE2N7 | 604 | 4 | 36.0% | 3 | 45.0% |
| isotig07096 | 604 | 65 | 100.0% | 109 | 100.0% |
| G8MPFOX02FTMH7 | 601 | 5 | 86.0% | 2 | 47.0% |
| G8KN99U01AMKWB | 599 | 178 | 100.0% | 288 | 99.0% |
| isotig02245 | 599 | 4 | 67.0% | 1 | 26.0% |
| isotig07204 | 599 | 6 | 85.0% | 1 | 16.0% |
| isotig07266 | 599 | 617 | 99.0% | 240 | 91.0% |
| G8KN99U01CXT3C | 596 | 2 | 50.0% | 3 | 55.0% |
| isotig07308 | 595 | - | 0.0% | 2 | 21.0% |
| G8KN99U02F9HPB | 594 | 122 | 99.0% | 72 | 96.0% |
| comp377322c0seq1 | 594 | 8 | 68.0% | 2 | 37.0% |
| isotig01817 | 593 | 1 | 29.0% | 3 | 41.0% |
| isotig02530 | 590 | - | 0.0% | 5 | 61.0% |
| G8MPFOX02I89FB | 589 | 8 | 86.0% | 17 | 88.0% |
| G8KN99U01C6INK | 586 | 4 | 30.0% | - | 0.0% |
| G8KN99U02H8A08 | 585 | 4 | 58.0% | 1 | 19.0% |
| isotig07487 | 583 | 3 | 39.0% | 4 | 32.0% |
| G8MPFOX02GPT01 | 582 | 7 | 46.0% | 11 | 55.0% |
| contig00454 | 582 | 3 | 74.0% | 3 | 52.0% |
| isotig07492 | 582 | 5 | 67.0% | 4 | 52.0% |
| isotig02854 | 580 | 1 | 30.0% | 9 | 81.0% |
| G8MPFOX01ED17U | 578 | 5 | 80.0% | 4 | 53.0% |
| G8MPFOX01AQL9I | 577 | 25 | 85.0% | 30 | 85.0% |
| comp423834c0seq1 | 574 | - | 0.0% | - | 0.0% |
| comp488293c0seq5 | 569 | 5 | 50.0% | 2 | 30.0% |
| comp411043c0seq1 | 567 | 32 | 33.0% | 53 | 67.0% |
| G8MPFOX02GS5MJ | 566 | 4 | 32.0% | 13 | 59.0% |
| comp487458c0seq1 | 566 | 4 | 75.0% | 7 | 63.0% |
| isotig07937 | 566 | - | 0.0% | 4 | 71.0% |
| G8KN99U02JOG92 | 564 | 1 | 35.0% | 2 | 56.0% |
| G8MPFOX02JIKAL | 564 | - | 0.0% | 2 | 46.0% |
| G8KN99U01EKO0K | 558 | 1 | 28.0% | - | 0.0% |
| isotig08062 | 558 | 1 | 32.0% | 6 | 63.0% |
| comp336759c0seq1 | 557 | - | 0.0% | 7 | 80.0% |
| isotig00556 | 557 | 1 | 30.0% | 1 | 18.0% |
| G8KN99U01CJIDW | 556 | 1 | 20.0% | 3 | 38.0% |
| comp338681c0seq1 | 556 | 5 | 64.0% | 7 | 78.0% |
| isotig02531 | 556 | 1 | 17.0% | 1 | 10.0% |
| G8MPFOX01CROME | 555 | - | 0.0% | - | 0.0% |
| isotig08151 | 553 | 266 | 99.0% | 314 | 100.0% |
| comp488808c0seq2 | 552 | 3 | 49.0% | 1 | 18.0% |
| G8KN99U02J2QHK | 548 | 1 | 29.0% | 5 | 57.0% |
| G8MPFOX01D98GC | 546 | 24 | 95.0% | 11 | 85.0% |
| isotig08315 | 544 | 8 | 45.0% | 9 | 92.0% |
| isotig01599 | 542 | 1 | 33.0% | 3 | 28.0% |
| isotig08351 | 539 | 1 | 21.0% | - | 0.0% |
| isotig08416 | 539 | 1 | 30.0% | 7 | 68.0% |
| G8MPFOX02GV8WC | 536 | 4 | 59.0% | 3 | 58.0% |
| G8MPFOX02F49BH | 529 | 3 | 34.0% | 5 | 90.0% |
| G8MPFOX02H6EOX | 529 | 3 | 40.0% | 4 | 62.0% |
| comp509852c0seq2 | 526 | 20 | 83.0% | 52 | 73.0% |
| G8KN99U01DQMYZ | 522 | 14 | 50.0% | 55 | 75.0% |
| isotig08718 | 522 | - | 0.0% | 3 | 63.0% |
| isotig03086 | 520 | 1 | 18.0% | 3 | 29.0% |
| comp520985c1seq1 | 516 | 5 | 47.0% | 1 | 29.0% |
| G8MPFOX01ASTBX | 513 | 106 | 99.0% | 50 | 100.0% |
| isotig02545 | 513 | 3 | 70.0% | 7 | 68.0% |
| isotig08856 | 512 | - | 0.0% | 4 | 26.0% |
| comp484170c0seq1 | 510 | 1 | 34.0% | 6 | 76.0% |
| isotig08881 | 510 | 1 | 33.0% | 5 | 67.0% |
| isotig08882 | 506 | - | 0.0% | - | 0.0% |
| contig01231 | 503 | 4 | 55.0% | 3 | 63.0% |

| absent in both | 12 (9%) |
| --- | --- |
| Warthog only | 6 (4%) |
| Bushpig only | 2 (1%) |
