# Supplementary material for: Functional analysis and transcriptional output of the Göttingen minipig genome
Source: BMC Genomics. 2015 Nov 14;16:932. doi: 10.1186/s12864-015-2119-7 (PMC4647470; doi:10.1186/s12864-015-2119-7)
Supplement: Additional file 11: Table S6. — Parameter settings for LC/MS analyte detection in positive or negative ion MRM mode. (DOCX 14 kb) [file 12864_2015_2119_MOESM11_ESM.docx]

**Additional file 11: Table S6**: parameter settings for LC/MS analyte detection in positive or negative ion MRM mode

| Analyte | Q1 Mass (m/z) | Q3 Mass (m/z) | Collision Energy (V) | Positive/Negative Ion MRM Mode |
| --- | --- | --- | --- | --- |
| 1’-hydroxymidazolam | 342.0 | 323.9 | 35 | + |
| benzydamine N-oxide | 326.1 | 102.0 | 29 | + |
| daunorubicinol | 530.2 | 383 | 19 | + |
| n-acetyl Sulfamethazine | 320.9 | 134 | 39 | + |
| SN-38 glucuronide | 569.1 | 393.0 | 45 | + |
| 8-oxo-O6-benzylguanine | 258.0 | 91.0 | 29 | + |
| hydroxybupropion | 256.2 | 238.2 | 19 | + |
| 4-hydroxydiclofenac | 312.0 | 230.4 | 40 | + |
| dextrorphan | 258.2 | 157.1 | 51 | + |
| hydroxytacrine | 215.1 | 197.0 | 25 | + |
| 4-hydroxytolbutamide | 285.2 | 186.0 | -24 | - |
| 7-hydroxycoumarin glucuronide | 337.1 | 160.9 | -36 | - |
| 7-hydroxycoumarin sulfate | 241.1 | 160.9 | -24 | - |
