# Supplementary material for: Functional analysis and transcriptional output of the Göttingen minipig genome
Source: BMC Genomics. 2015 Nov 14;16:932. doi: 10.1186/s12864-015-2119-7 (PMC4647470; doi:10.1186/s12864-015-2119-7)
Supplement: Additional file 6: Figure S2. — Sequence analysis of the HEPN1 pseudogene. Alignment of the human HEPN1 mRNA sequence (NM_001037558) with orthologs from the minipig, the Duroc Pig, Phacochoerus africanus (Warthog) and Potamochoerus larvatus (Bushpig. The A/G start-codon mutation is highlighted in green, insertions or deletions are in yellow, stop-codons in red and conserved sequences are colored in magenta. (PDF 115 kb) [file 12864_2015_2119_MOESM6_ESM.pdf]

```
                                                                     |-> start
HUMAN    GCAAACTAGAAATGTCAGTGCCTTTGGGGCTGAGACAAAACTTGACCTGGTGTGGAGGTGATGGGTAACT
PIG      GCCAATCAGAAACGTTGTTGCCTTTGGGGCTGGGACAAAACTTGACCCAGTGTGGA.ATAGTGGGGAACA
MINIPIG  GCCAATCAGAAACGTTGTTGCCCTTGGGGCTGGGACAAAACTTGACCCAGTGTGGA.ATAGTGGGGAACA
BUSHPIG  GCCAATCAGAAACGTTGTTGCCTTTGGGGCTGGGACAAAACTTGACCCAGTGTGGA.ATAGTGGGGAACA
WARTHOG  GCCAATCAGAAACGTTGTTGCCCTTGGGGCTGGGACAAAACTTGACCCAGTGTGGA.ATAGTGGGGAACA

HUMAN    GGGGCCTTGGAATTGCTCCATGGGTTGATGGCGAATCAGAGCTGGAGTTTAGGAGACTAGGGATG...CA
PIG      G.......CACAGTGCTCTGTGGGATGGTACAGAGTCAGAGCTGGAGCTTAGGAAGCTGGGGAGGGGCCA
MINIPIG  G.......CACAGTGCTCTGTGGGATGGTACAGAGTCAGAGCTGGAGCTTAGGAAGCTGGGGAGGGGCCA
BUSHPIG  G.......CACAGTGCTCTGTGGGATGGTACAGAGTCAGAGCTGGAGCTTAGGAAGCTGGGGAGGGGCCA
WARTHOG  G.......CACAGTGCTCTGTGGGATGGTACAGAGTCAGAGCTGGAGC.TAGGAAGCTGGGGAGGGGCCA

HUMAN    AGGAC.CCTTGGAGGCATTAAGGAGGAGGGAATGGAATACACAGAGGGCCTCCTTCTCTTTCAGCTTTTT
PIG      AGGATGCCCTAGAGGCATCAGGGAAGAGAAGTCAGAACACACAGAGGGACTCCTTTACTTTCAGTTTTTT
MINIPIG  AGGATGCCCTAGAGGCATCAGGGAAGAGAAGTCAGAACACACAGAGGGACTCCTTTACTTTCAGTTTTTT
BUSHPIG  AGGATGCCCTAGAGGCATCAGGGAAGAGAAGTCAGAACACACAGAGGGACTCCTTTACTTTCAGTTTTTT
WARTHOG  AGGATGCCCTAGAGGCATCAGGGAAGAGAAGTCAGAACACACAGAGGGACTCCTTTTCTTTCAGTTTTTT

HUMAN    AATTGCCCTCTCTCCTCACACAGTAGATTACTGCCACTCCTATGAACTGTTCAATAGGCGGTGGCATGGG
PIG      AACTGCCCTCTCTCCTCACACAGTAGATTATTGTCTTTCCTACAAACTGTTCAAGAGGCGGTGGCATGGG
MINIPIG  AACTGCCCTCTCTCCTCACACAGTAGATTATTGTCTTTCCTACAAACTGTTCAAGAGGCGGTGGCATGGG
BUSHPIG  AACTGCCCTCTCTCCTCACACAGTAGATTATTGTCTTTCCTACAAACTGTTCAAGAGGCGGTGGCATGGG
WARTHOG  AACTGCCCTCTCTCCTCACACAGTAGATTATTGTCTTTCCTACAAACTGTTCAAGAGGCGGTGGCATGGG

                                                      stop <-|
HUMAN    CATGTCCTGGCTACACAGCGGCCCAGCCTCTT..TATTTTGATGTTAGTGTGATTAGGGAGTCTGCCCTT
PIG      CATGGCCTAGCCACACAGTGGACCAACCATTTGGCATTTTGATGTTTGTGTGAATAGGGAGTCTGCACTT
MINIPIG  CATGGCCTAGCCACACAGTGGACCAACCATTTGGCATTTTGATGTTTGTGTGAATAGGGAGTCTGCACTT
BUSHPIG  CATGGCCTAGCCACACAGTGGACCAACCATTTGGCATTTTGATGTTTGTGTGAATAGGGAGTCTGCACTT
WARTHOG  CATGGCCTAGCCACACAGTGGACCAACCATTTGGCATTTTGATGTTTGTGTGAATAGGGAGTCTGCACTT
```

Figure S2
